# ILIVE Project Volunteer study. Developing international consensus for a European Core Curriculum for hospital end-of-life-care volunteer services, to train volunteers to support patients in the last weeks of life: A Delphi study

**DOI:** 10.1177/02692163211045305

**Published:** 2021-10-20

**Authors:** Tamsin McGlinchey, Stephen R Mason, Ruthmarijke Smeding, Anne Goosensen, Inmaculada Ruiz-Torreras, Dagny Faksvåg Haugen, Miša Bakan, John E Ellershaw

**Affiliations:** 1 Palliative Care Unit, University of Liverpool, Liverpool, UK; 2 University of Humanistic Studies, Utrecht, The Netherlands; 3 Cudeca Hospice Foundation, Malaga, Spain; 4 Department of Clinical Medicine K1, University of Bergen, Bergen, Norway; 5 Regional Centre of Excellence for Palliative Care, Haukeland University Hospital, Bergen, Norway; 6 University Clinic of Pulmonary and Allergic Diseases Golnik, Golnik, Slovenia

**Keywords:** End of life, palliative care, volunteers, hospital, consensus, Delphi

## Abstract

**Background:**

Volunteers make a huge contribution to the health and wellbeing of the population and can improve satisfaction with care especially in the hospice setting. However, palliative and end-of-life-care volunteer services in the hospital setting are relatively uncommon. The iLIVE Volunteer Study, one of eight work-packages within the iLIVE Project, was tasked with developing a European Core Curriculum for End-of-Life-Care Volunteers in hospital.

**Aim:**

Establish an international consensus on the content of a European Core Curriculum for hospital end-of-life-care volunteer services which support patients in the last weeks of life.

**Design:**

Delphi Process comprising the following three stages:

1. Scoping review of literature into palliative care volunteers.

2. Two rounds of Delphi Questionnaire.

3. Nominal Group Meeting.

**Setting/participants:**

Sixty-six participants completed the Round 1 Delphi questionnaire; 75% (50/66) took part in Round 2. Seventeen participants attended the Nominal Group Meeting representing an international and multi-professional group including, clinicians, researchers and volunteer coordinators from the participating countries.

**Results:**

The scoping review identified 88 items for the Delphi questionnaire. Items encompassed organisational issues for implementation and topics for volunteer training. Three items were combined and one item added in Round 2. Following the Nominal Group Meeting 53/87 items reached consensus.

**Conclusion:**

Key items for volunteer training were agreed alongside items for implementation to embed the end-of-life-care volunteer service within the hospital. Recommendations for further research included in-depth assessment of the implementation and experiences of end-of-life-care volunteer services. The developed European Core Curriculum can be adapted to fit local cultural and organisational contexts.

**What is already known about the topic?**

- Despite a majority of people expressing a wish to die at home, many people will still die in hospital and have limited access to support of volunteers.
- Palliative Care Volunteers have been shown to improve care and provide valuable support to patients and families
- There is no standardised training programme for hospital-based volunteers who support dying patients, and equally no empirical data of their effect in this clinical setting

**What this paper adds?**

- A total of 53 items deemed to be essential for the development, training and implementation of hospital end-of-life-care volunteer services which support patients in the last weeks of life were identified from the Delphi study.
- Results from the Delphi informed the development of a European Core Curriculum addressing service implementation and specific training for development of hospital end-of-life-care volunteer services to support patients in the last week of life.

**Implications for practice, theory or policy?**

- The European Core Curriculum provides a framework to design, develop and implement hospital end-of-life-care services, to support patients in the last weeks of life.
- Establishing a European Core Curriculum will enable policy makers to consider stratified approaches to service delivery, and provide a potential framework for benchmarking for hospital end-of-life-care volunteer services.
- Research as part of iLIVE Volunteer Study will provide important information on end-of-life-care volunteers within the hospital setting, across five countries in Europe.

## Background

The need for palliative care is increasing globally due to a rise in population and overall life expectancy, bringing with it a higher prevalence of chronic illness.^1^ This rise in demand will challenge the capacity of healthcare systems to provide access to palliative care and psychosocial support, requiring innovations to meet population needs.^2^ This will be particularly important for hospitals as, despite an increasing number of people expressing a wish to die at home, the number of patients dying in hospital will remain significant.^3^ Volunteers have traditionally played a positive and significant role in the delivery of palliative care services, especially in hospice care,^4^ therefore, further development of volunteer services may be one such innovation to optimise the delivery of palliative care in the hospital setting. Volunteers give time, skills and expertise freely and contribute millions of hours of work making a huge contribution to the health and wellbeing of the population.^5^ The involvement of volunteers in the hospice setting has been shown to have an impact on a number of areas including improving levels of satisfaction with care^6,7^ and even increase survival time.^7,8^ Volunteers also provide a much-needed community resource through offering social support, fulfilling surrogate family roles and even ‘mediation’ between patients and staff.^9,10^

It has been suggested that hospital is a less than optimal setting for the delivery of palliative care, or for dying patients,^11^ and media reports have attributed dying away from home, in hospital, as an indicator of neglect or a lack of care.^12,13^ These perceptions have shaped national policies for palliative care, helping to ‘problematise’ hospital as a place of care for dying patients.^14^ More recent evidence challenges this perspective, highlighting that hospitals can confer benefit for many palliative care patients, offering a place of ‘safety’, particularly for patients with cancer and patients from deprived backgrounds.^15^ It may be that the development of hospital based volunteer services has the potential to enhance the experience of dying patients in hospital, particularly for those concerned with being alone, through the practice of ‘being there’^16–18^ or by providing additional emotional or psychosocial support as an independent person.^7,18^ Volunteer services may introduce a valuable sense of ‘community’ back into the hospital environment, which could lead to important improvements in care of the dying.

There remains a lack of robust evidence regarding how best to train and support palliative care volunteers,^19^ especially in the care of dying patients. More specifically, a recent systematic review and narrative synthesis revealed the unique training and support needs of hospital based palliative care volunteers due to the complex, ever changing and highly structured environment within which they are supporting patients.^18^ Ensuring that hospital palliative and end of life care volunteers are appropriately prepared through training has the potential to increase satisfaction with their role as well as aid in retention of volunteers within the service.^20,21^ Evidence also suggests there is an appetite for wider sharing of training objectives, materials and procedures to ensure greater transparency of the development of volunteer services across international boundaries.^22^

The iLIVE Project, an European Union Horizon 2020 funded study, aims to address these concerns.^11^ The iLIVE Volunteer Study, one of eight work-packages within the iLIVE Project, was tasked with developing a European Core Curriculum for end-of-life-care Volunteers in the hospital setting who support patients in the last weeks of life. Due to a lack of existing evidence, and heterogeneity of existing volunteer services, especially in the hospital setting, a Delphi study was undertaken to gain international consensus on what should be included within the European Core Curriculum for end-of-life-care volunteer services.

### Aim

Establish an international consensus on the content of a European Core Curriculum for hospital end-of-life-care volunteer services for patients in the last weeks of life.

## Methodology

Delphi studies have been used widely to drive the development of best practice guidelines in palliative care, providing a consensus building approach to the collection and synthesis of data. Delphi gathers informed opinions from a group of experts who are knowledgeable in a specialised area^23^ to formulate a consensus when there is a paucity of evidence, or the field of exploration is new and uncharted.^24,25^ The guidance for conducting and reporting Delphi studies^26^ was used to ensure robust method and reporting.

The Delphi process comprised three stages:

1. Undertake a scoping review of available literature on palliative and end-of-life-care volunteering to identify key concepts/themes for service implementation and volunteer training with which to develop items for the Delphi questionnaire;
2. Conduct two rounds of the Delphi Questionnaire to assess levels of agreement for each item;
3. Using Nominal Group Technique, gain ‘Consensus Agreement’ on included items.

Each stage had a specific method and approach, and was undertaken consecutively to inform the process for the next stage. The following sections describe the process undertaken for each stage.

### Stage 1: Scoping review: Key concepts/themes for the development of the Delphi questionnaire

Following the five-stage framework approach by Arskey and O’Malley,^27^ a scoping review was undertaken to identify items for inclusion in the Delphi questionnaire. Using definitions by Munn et al.,^28^ a scoping review was appropriate as the purpose was to ‘identify key characteristics or factors’^28^ related to service implementation, education and training of volunteers, to support patients in hospital at the end of life. Specifically, the output of this review is a list of items that will be included in the first round of the Delphi questionnaire..

Database Search: The scoping review was conducted using a structured search using the Scopus tool which searches over 14,000 Scientific, Technical and Medical and Social Science publications including Embase and Medline.

Search Criteria: We used a ‘search string’ first developed in the EU funded study, OPCARE9, as part of a specific work-package to scope the types and breadth of palliative care volunteer services for cancer patients at the end of life, across Europe.^29^ Key terms included ‘palliative care’, ‘dying’, ‘end of life care’, ‘volunteers’ and ‘informal carers’. The inclusion criteria was intentionally broad, engaging all study types. The OPCARE9 review concluded in 2009, therefore, this study looked to capture articles that had been published since the end of this study. We focussed on articles published in English, between January 2009 and February 2019. Due to the large number of articles retrieved, as well as the time constraints of the project, the search was not expanded to other databases, grey literature or hand searching.

The protocol for the scoping review has been included in the Supplemental Material alongside this article.

Creation of ‘themes’: Articles were deemed relevant for inclusion if they contained a critical examination or exploration of issues specific to volunteering in palliative and end of life care. Articles were reviewed and contents were coded to develop a thematic framework^30^ based on the main outcomes/discussion reported. Articles were examined for information on:

- Volunteer roles in caring for patients and their families;
- Volunteer roles in the wider care providing organisation;
- Education and training, including organisational regulations and supervision;
- Support for volunteers working in palliative and end of life care.

Sections of article text that were related to the review question were coded by one of the study researchers (TM). The purpose of the codes were to label the text so that salient information related to the review question could be identified. Codes aimed to identify key characteristics or factors related to service implementation, education and training of volunteers. Codes from across all included articles were then reviewed, and the codes were categorised to create themes. Themes from the review were used to structure the creation of items for the Delphi questionnaire, to represent the key characteristics or factors related to the individual theme. The development of individual items for the Delphi questionnaire was undertaken by three members of the research team (AG, SM and TM) to allow further discussion and refinement, and to develop a comprehensive list for inclusion in the Delphi questionnaire. The final list of items included in the Delphi questionnaire can be found in the Supplemental Materials provided with this article.

Results are reported using the Preferred Reporting Items for Systematic reviews and Meta-Analyses extension for Scoping Reviews checklist extension for scoping studies.^31^

### Stage 2: Delphi questionnaire

The Round 1 Delphi questionnaire was developed from the findings of the Scoping Review, and constructed using Google Forms; an easily accessible online format to promote greater participation. Data were collected between March and May 2019.

#### Ethical review

Approval for the Delphi Questionnaire was given by the University of Liverpool Ethics Committee (reference number: 4959, 17th March 2019).

#### Sampling

Convenience and Snowball sampling was used to recruit participants.^32^ The questionnaire was sent to members of the iLIVE Consortium with expertise in volunteering, palliative and end-of-life care, including multi-disciplinary healthcare professionals and educators (convenience sampling). The iLIVE Consortium also included volunteer management/coordinators linked to the project. Initial recipients of the questionnaire were encouraged to forward the questionnaire to appropriate contacts whom they felt may have an interest in the project (snowball sampling), including the EAPC Task Force for Hospice and Palliative Care Volunteering.

#### Consent to participate

All potential participants were provided with an information sheet and were asked to complete an electronic consent form. Distribution and return of questionnaires was anonymous from other participants, promoting ‘independent objectivity’.^33^ To ensure confidentiality, questionnaires were given a unique identifier, with the link between the identifier and participant destroyed on completion of round two.

#### Delphi rounds

The Round 1 Delphi Questionnaire contained 88 items. Participants were asked to rate their level of agreement that the item should be included in the European Core Curriculum (5-point Likert scale: 1 = strongly disagree; 5 = strongly agree). The questionnaire also collected ‘free text’ comments against all items (in both Round 1 and Round 2). Results from Round 1 were used to inform the questionnaire for Round 2, which repeated the process.

For Round 2 participants were provided with a breakdown of their individual responses from Round 1, alongside the aggregated group response.

#### Data analysis

Percentages, median values and inter quartile ranges (IQRs) were calculated for each item from Rounds 1 and 2 to describe the spread of answers and compare results between rounds. These values (percentage, median and IQR) were used to determine the ‘level of agreement’ across participants, for each ‘item’^34^:

- ‘Very high agreement’ – median 5; percentage agreement ⩾80%; IQR 0.
- ‘High agreement’ – median 4/5; percentage agreement ⩾80%; IQR 1.
- ‘Moderate agreement’ – median ⩽4; percentage agreement 60%–79%; IQR 1.
- ‘Low agreement’ – median <4; percentage agreement <60%; IQR >1.

### Stage 3: International consensus agreement: Nominal group technique

Guzys et al.^35^ proposes that the Delphi process is inherently interpretivist in nature, promoting reflection through an iterative and cyclical process. Considering the constructivist nature of the Delphi process this study does not assume that ‘agreement’ (as defined by the quantitative analysis) implies a ‘correct’ answer or judgement.^26^ Building on the iterative process, and being sensitive to the position that Delphi should not transform subjective opinion into objective data,^35^ this aspect of the study brought together participants who completed the Delphi questionnaire, along with members of the iLIVE Consortium who have a specific focus on end-of-life-care, in a final face-to-face meeting using Nominal Group Technique.^36,37^ The purpose was to enable individual reflection on the results from the two Delphi rounds, as well as provide an opportunity for group discussion. The implicit or ‘tacit’ knowledge from the expert panel was integral to generating the final list of items. Participants were able to question, reflect and discuss individual items and ratings from the Delphi questionnaire to facilitate ‘sharing of perspectives to create new knowledge’,^35^ with the specific aim of generating a list of essential items for inclusion in a core curriculum, to train end-of-life-care volunteers in the hospital setting.

The Nominal Group Technique included three separate discussion topics, based on the levels of agreement from the Delphi questionnaire; low agreement items, very high agreement items and moderate/high agreement items. Participants were split into two smaller groups to promote easier discussion, and a facilitator guided each group through the three discussion topics. Each discussion topic had the following structure:

1. Silent generation of ideas: individual reflection on items and ratings;
2. Round Robin gathering of questions/thoughts/ideas: each group member was given the opportunity to feedback their thoughts on the items being discussed;
3. Open group discussion: group discussion based on round robin;
4. Generation of statements from group discussion: to reflect the discussions/decisions, for feedback to the wider group.

Following all three discussion topics, participants were brought together for consolidation and final consensus. Discussion of all items took place and all members agreed whether an item should be included or not. In the event of any disagreement, a vote was taken to establish consensus for that item.

## Results

The main findings from all three stages are presented in this section.

### Stage 1: Scoping review

#### Included articles

**Figure 1.**
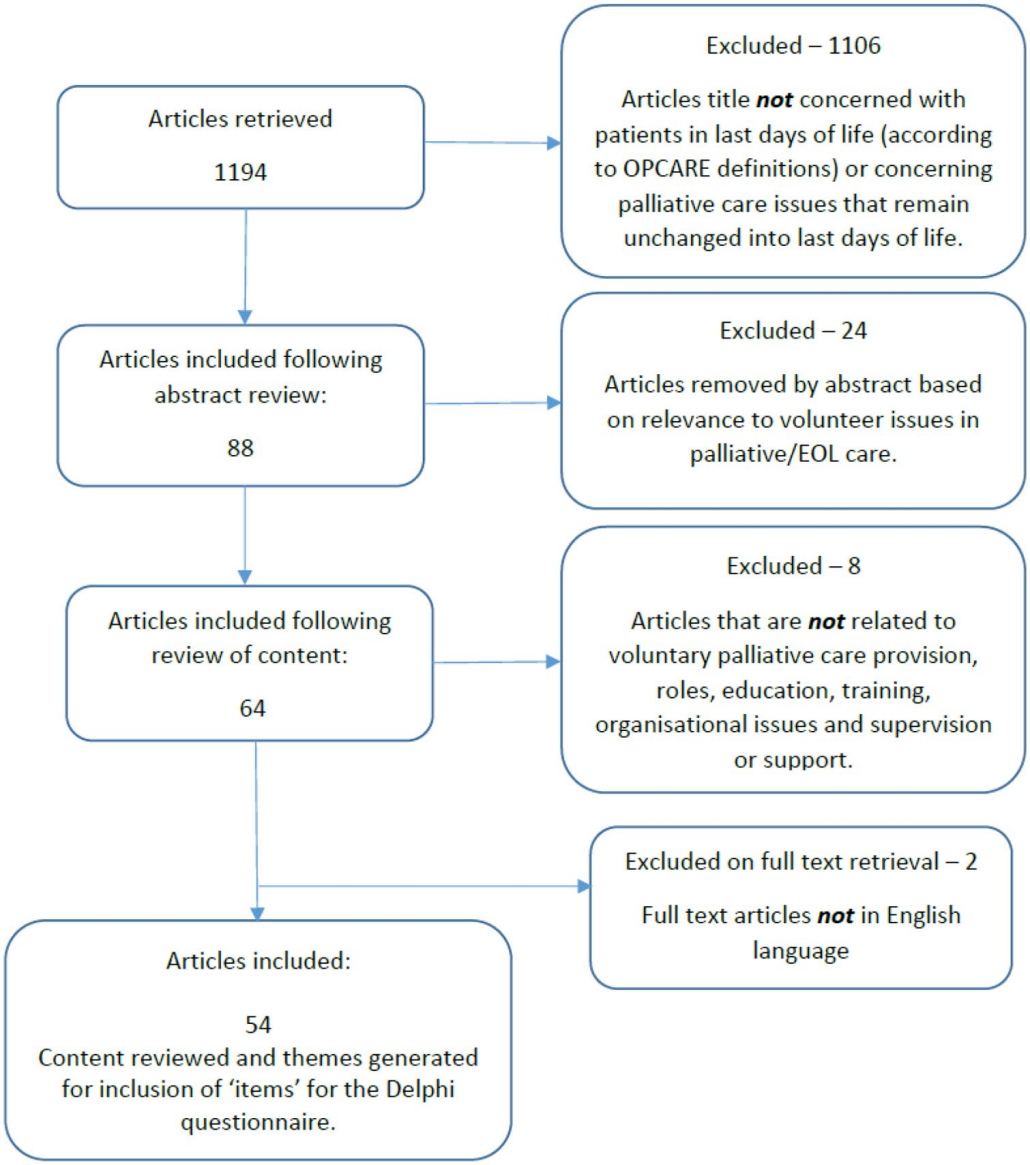
Scoping review flow diagram.

A total of 1194 articles met the initial search criteria following application of the search string (Figure 1). The content of the 54 articles identified as relevant for the study was analysed to identify ‘items’ for the Delphi questionnaire (Table 1).

**Table 1.** Individual items included in Delphi questionnaire, including breakdown of responses per item – Delphi Round 1/Round 2.

Individual items on Delphi questionnaire		Round 1				Round 2			
		Median rating (*n* = 66)	IQR	% Agreement (4/5 on scale)	Level of agreement	Median rating (*n* = 50)	IQR	% Agreement (4/5 on scale)	Level of agreement
Section 1: ‘being there’ and ‘being present’ with the patient/family									
1a	Establishing an environment of ‘mutuality’ to promote empathy and a ‘non-judgemental’ relationship	5	0	97	Very high	5	1	100	Very high
1b	Being attentive to the emotional needs of persons at the end of life (e.g. listening to the patient’s/family’s fears, worries, hopes, dreams, other feelings, etc.)	5	0	97	Very high	5	0	98	Very high
1c	Being responsive to the ‘uniqueness of the other’	5	1	88	High	5	1	92	High
1d	How to be ‘present’ with patients and families	5	0	100	Very high	5	0	96	Very high
1e	Relational attunement; establishing a connection, building rapport and relationship building with patients and families	5	1	91	High	5	1	90	High
1f	Journeying with patients, sitting with patients in the last hours of life	4	1	82	High	5	1	86	High
1g	Providing social support to patients and their families (e.g. talking with patients/families, sharing hobbies and interests, reading to the patient, etc.)	4	1	79	Moderate	4	1	90	High
1h	Understanding the social nature of the volunteer role	5	1	88	High	5	1	96	High
1i	Use of humour in patient/volunteer interactions	4	1	64	Moderate	4	1	72	Moderate
Section 2: communication skills									
2a	Advanced communication skills training: listening skills and responding to patient and family emotions	5	1	89	High	5	0	92	Very high
2b	Advanced communication skills training: barriers to effective communication	5	1	91	High	5	1	92	High
2c	Understanding of ‘Do Not Attempt Resuscitation’ orders, living wills and power of attorney	4	2	58	Low	4	2	60	Low
2d	Communication with patients with dementia and cognitive decline	5	1	92	High	5	1	90	High
2e	Communication skills for talking with children	4	2	73	Low	4	1	80	High
2f	Understanding issues of denial, including when and how to address this with the patient’s care team	4	2	71	Low	4	2	70	Low
2g	Understanding issues of collusion, including when and how to address this with the patient’s care team	4	2	64	Low	4	1	76	Moderate
2h	General Introduction to Communication Skills: the need for good communication skills	5	0	53	Very high	5	0	94	Very high
2i	Communication skills to support conversations around future care planning (Advance Care Planning)	4	2	53	Low	4	2	58	Low
2j	Communication skills to support conversations around end-of-life care issues	4	1	80	High	4	1	82	High
Section 3: cultural competency									
3a	Understanding diversity and seeing patients and their families as individuals	5	1	92	High	5	0	96	Very high
3b	Understanding personal values, belief systems, attitudes, judgements and worldviews and how these may impact on the care and support provided to patients and their families	5	0	94	Very high	5	1	94	High
3c	How to support patients with diverse cultures, values, beliefs and feelings	5	1	94	High	5	1	82	High
3d	Peer Support: Activities/resources to build and facilitate strong relationships with other volunteer colleagues, to discuss difficult situations or patients, to ask questions and give or receive advice in a friendly non-judgemental environment	5	1	95	High	5	1	94	High
3e	Understanding behaviours related to fears around death and dying, including ‘fear of death’ and ‘death anxiety’	5	1	85	High	5	1	94	High
Section 4: end of life phenomena									
4a	Knowledge and understanding of different end of life phenomena	4	1	79	Moderate	4	2	74	Low
4b	Understanding the prevalence and impact of end of life phenomena on patients	4	2	74	Low	4	2	66	Low
4c	How to offer support to patients and families regarding end of life phenomena	4	1	89	High	4	1	80	High
Section 5: defining and promoting understanding of the volunteer role									
5a	Understanding the ‘definition’ of the volunteer role within the service	5	0	94	Very high	5	0	96	Very high
5b	Understanding the volunteer role as part of the care team	5	0	98	Very high	5	0	100	Very high
5c	Understanding of the complexities of the care environment and the role of the volunteer within it; exploring power relationships between volunteer/staff and volunteer/patient and family	5	1	88	High	5	1	98	High
Section 6: ethical issues relating to end of life care and the volunteer role									
6a	Issues of confidentiality and how to navigate this within the volunteer role	5	0	95	Very high	5	0	98	Very high
6b	How to ensure confidentiality is upheld whilst undertaking the volunteer role	5	0	95	Very high	5	0	96	Very high
6c	Negotiating ‘boundary spaces’ within the role of a volunteer (e.g. not ‘friend’ or ‘professional’ and not ‘paid’ member of the organisation)	5	1	92	High	5	1	92	High
6d	Understanding of ethical Issues that could be encountered as part of the volunteer role (e.g. ethical dilemmas, competing interests, receiving gifts, clinical concerns, etc.)	5	1	92	High	5	1	96	High
6e	Understanding ethical issues in palliative and end of life care (e.g. assisted suicide, hastening death, etc.)	5	1	82	High	4	1	84	High
6f	Dealing with experiences of ‘powerlessness’ within the volunteer role, avoiding burnout and promoting resilience (e.g. often the volunteer role is to ‘be there’ with patients and families rather than actively ‘doing’ for them, leaving the potential to feel ‘powerless’ and frustrated with help they can offer)	New R2				5	0	100	Very high
Section 7: loss, grief and bereavement									
7a	Understanding processes of loss	5	1	97	High	5 (7a–c)	1	94	High
7b	Understanding processes of grief	5	1	97	High	7a–7c merged R^2^			
7c	Understanding processes of bereavement	5	1	91	High				
7d	Learning how to provide support to families, through grief and bereavement	5	1	85	High	5	1	88	High
7e	Understanding the nature and impact of ‘Complicated Grief’	4	2	68	Low	4	2	74	Low
7f	Exploring personal experiences of grief and how this may impact the volunteer in their role	5	1	97	High	5	1	98	High
Section 8: physical signs and symptoms in palliative and end of life care									
8a	Prepare the volunteer for naturally occurring changes in the patient towards the end of life, including how to communicate this to family members	4	2	71	Low	4	1	88	High
8b	Issues relating to patients in isolation due to disease/condition	4	2	65	Low	4	2	70	Low
8c	Understanding common symptoms at the end of life	4	1	83	High	4	1	88	High
8d	Understanding the physical needs of persons at the end of life (e.g. mobility, cognition, dysphasia, etc.)	4	1	85	High	4	1	80	High
8e	Understanding of issues of hydration at the end of life	4	2	70	Low	4	2	68	Low
8f	Understanding of issues of nutrition at the end of life	4	2	65	Low	4	2	68	Low
8g	Understanding of issues of artificial hydration at the end of life	4	2	50	Low	3	2	48	Low
8h	Understanding of issues of artificial nutrition at the end of life	3	3	47	Low	3	2	46	Low
8i	Understanding of common medications used for pain and symptom control	3	1	44	Low	4	1	54	Low
8j	Caring for ‘actively dying’ patients (e.g. days/hours leading up to death)	4	1	77	Moderate	4	1	80	High
8k	Understanding of the physiology, signs and symptoms, of dying	4	2	74	Low	4	1	84	High
Section 9: practical aspects of the volunteer role (delivering care and support)									
9a	Comfort measures and strategies to support the patient (e.g. relaxation techniques, meditation, music/art therapy)	4	2	74	Low	4	1	78	Moderate
9b	‘Hands on’ comfort measures to provide comfort to the patients (e.g. touch, massage)	4	2	74	Low	4	2	74	Low
9c	Establishing a process of ‘handover’ between volunteers to support continuity of care	4	1	82	High	4	1	84	High
9d	Providing practical support to patients and their families (e.g. running errands and responding to needs)	4	1	79	Moderate	4	1	78	Moderate
9e	Identification of patients/family in need of volunteer support	4	1	82	High	4	2	74	Low
9f	Practical care that can be delivered by the bedside (e.g. helping with eating, drinking, support with washing and cleaning teeth, etc.)	4	2	73	Low	4	1	76	Moderate
Section 10: psychological/psychosocial aspects of care at the end of life									
10a	Issues regarding depression at the end of life	4	2	70	Low	4	1	68	Moderate
10b	Issues regarding anxiety at the end of life	4	1	76	Moderate	4	2	70	Low
10c	Being able to recognise when patients might be suicidal and how to address this with the patients’ care team	3	2	41	Low	4	2	58	Low
10d	Understanding techniques and strategies for dealing with aggression (patients/families/other)	4	2	71	Low	4	1	82	High
10e	Family dynamics (e.g. mediating, dealing with conflict)	4	2	67	Low	4	2	70	Low
Section 11: religion and spirituality									
11a	Understanding the difference between religious and spiritual needs	5	1	89	High	5	1	86	High
11b	Understanding and acceptance of, and respect for, the spiritual needs of persons at the end of life	5	1	94	High	5	0	96	Very high
11c	Understanding spiritual diversity	5	1	89	High	5	1	96	High
11d	Being aware of religious/spiritual needs of patients and their families, and being able to ‘signpost’ for further support if required	4	2	61	Low	4	1	82	High
Section 12: volunteer as patient/family advocate									
12a	How to provide advocacy support for patients and their families	4	2	59	Low	4	2	70	Low
12b	Understanding patient rights	4	1	76	Moderate	5	1	88	High
12c	Being a source of informational support to patients and their families	4	2	59	Low	4	2	72	Low
Section 13: volunteer recruitment/retention									
13a	Use of ‘motivation’ (to be a volunteer) assessment tool as part of the volunteer selection process	5	2	73	Low	4	2	74	Low
13b	Use of a ‘personality’ assessment tool as part of the selection process	5	2	70	Low	4	2	56	Low
Section 14: volunteer support									
14a	Self-care information and strategies and personal resilience	5	1	94	High	5	1	98	High
14b	Regular ongoing mentoring	5	1	92	High	5	1	96	High
14c	Personal Death Awareness	4	1	79	Moderate	4	1	86	High
14d	Rituals in dying: practising ‘rituals’ and other ways to honour the lives of patients	4	1	61	Moderate	4	1	76	Moderate
14e	Establish an environment for informal supervision/formal structured supervision with feedback	5	1	88	High	5	1	96	High
14f	Coping strategies for dealing with suffering and death	5	1	94	High	5	1	96	High
14g	Access to wider support services and Psychological support	4	2	65	Low	4	1	76	Moderate
14h	Training updates and other ongoing educational opportunities	5	1	86	High	5	1	92	High
Section 15: community engagement and advocacy for the volunteer programme									
15a	How to engage with community outreach opportunities within the local community to raise awareness of the volunteer programme	4	1	76	Moderate	4	2	72	Low
15b	Engaging with staff and management within the care providing organisation, to promote the work of the volunteer service	4	1	83	High	4	1	76	Moderate
Section 16: volunteer competency and volunteer assessment									
16a	Development of ‘Core Competencies’ for volunteers providing support to patients in the last days of life, and their families	5	1	94	High	5	1	96	High
16b	Development/agreement of ‘standard’ outcome measures to evaluate benefit of the programme	4	1	77	Moderate	5	1	82	High
16c	Include ‘Formative Assessment’ of volunteers following training programme	5	1	76	Moderate	4	1	76	Moderate
16d	Include ‘Summative Assessment’ of volunteers following training programme	4	2	68	Low	4	2	72	Low
Section 17: issues of organisational infrastructure and implementation									
17a	Embed the volunteer service within the organisation, with attention to organisational/regional/national/international Legislation affecting volunteers	5	1	85	High	5	1	90	High
17b	Establish organisational policy and procedures for role of the volunteer service and volunteer coordinator	5	1	94	High	5	1	96	High

### Stage 2: Delphi rounds

#### Participation

Table 2 shows participation in the two rounds of the Delphi questionnaire by country, age and gender. Participants held the following areas of expertise: Current Volunteers – palliative care and general; Volunteer Service Management/Volunteer Coordinators; Medical experts in Palliative Medicine; Nurse experts in Palliative Care; Social Research (Psychologists, Sociologists, Humanistic Studies); Educationalists; Law – including ethics and palliative care; Social Workers, including Social Workers in Palliative Care.

**Table 2.** Participation in the two rounds of Delphi questionnaire by country, age and gender.

Participation per continent	Number of participants	
	Round 1	Round 2
Europe (Austria, Belgium, Spain, France, Germany, Iceland, The Netherlands, Norway, Poland, Serbia, Slovenia, Sweden and Switzerland)	38	32
United Kingdom*	14	9
South America (Argentina and Brazil)	10	7
Asia (India and Pakistan)	2	1
Oceania (New Zealand)	1	1
Africa (Uganda)	1	0
Total	66	50
Age and gender	Round 1	Round 2
Age		
Median	55	57
Range (Min–Max)	28–72	28–72
	*n* = 65**	*n* = 49**
Gender		
Female	74% (*n* = 48/66)	74% (*n* = 37/50)
Profession	Round 1	Round 2
Palliative care physician	16	14
Palliative care nurse	8	5
Volunteer service management/co-ordinator	6	6
Social research (psychologists, sociologists and humanistic studies)	6	4
Educationalist	6	3
Physician (other speciality)	5	3
Hospice director/CEO	4	3
Volunteer (palliative care)	4	3
Social worker	4	5
Nurse (other speciality)	3	2
Other***	3	2

* Due to a high number of participants, the United Kingdom is listed separately.

** 1 participant entered ‘>18’ instead of numerical figures into the age field on both Round 1 and 2 questionnaires precluding their response from being included in the median age calculation.

*** Other includes: social worker (palliative care); spiritual leader; palliative care charity director.

#### Round 1 Delphi questionnaire results

Table 1 illustrates the level of agreement received for each item in Round 1 and Round 2. Overall 97% (85/88) of items had a median priority rating of 4 or 5, indicating that most participants thought the items listed for inclusion were important. Around two thirds of the individual items (64%, 56/88) achieved a ‘*Very High*’ or ‘*High*’ level of agreement for inclusion. Table 3 shows amendments following free text comments received following completion of the Round 1 questionnaire. Due to variations in the level of ‘agreement’ across many of the items, it was important that participants were able to review their responses in the second round of the Delphi.

**Table 3.** Amendments to Round 2 Delphi questionnaire, following free text comments from Round 1.

Original	Amended wording for Round 2
2 (f) Dealing with issues of denial	2 (f) Understanding issues of denial, including when and how to address this with the patients care team
2 (g) Dealing with issues of collusion	2 (g) Understanding issues of collusion, including when and how to address this with the patient’s care team
3 (e) Exploration of fear of death and death anxiety	3 (e) Understanding behaviours related to fears around death and dying, including ‘fear of death’ and ‘death anxiety’
7 (a) Understanding loss	Comments indicated that these three concepts were not separate in many languages, so they were combined for round two: 7 (a) Understanding processes of loss, grief and bereavement
7 (b) Understanding grief	
7 (c) Understanding bereavement	
7 (f) Exploring the personal impact of grief and impact on the volunteer role	7 (f) Exploring personal experiences of grief and how this may impact the volunteer in their role
8 (a) Recognising changes in a patient’s clinical condition	8 (a) Prepare the volunteer for naturally occurring changes in the patient towards the end of life, including how to communicate this to family members
10 (c) Understanding techniques and strategies for dealing with suicidal patients	10 (c) Being able to recognise when patients might be suicidal and how to address this with the patients’ care team
11 (d) Providing religious/spiritual support to patients and their families	11 (d) Being aware of religious/spiritual needs of patients and their families, and being able to ‘signpost’ for further support if required
*Additional* item included in Round 2 following comments from Round 1	
6 (f) Dealing with experiences of ‘powerlessness’ within the volunteer role, avoiding burnout and promoting resilience (e.g. often the volunteer role is to ‘be there’ with patients and families rather than actively ‘doing’ for them, leaving the potential to feel ‘powerless’ and frustrated with help they can offer)	

#### Round 2 Delphi questionnaire results

Following analysis of Round 1, 87 individual items were included in the questionnaire (see Table 3 for Delphi questionnaire amendments). As in Round 1 just under two thirds of items (54/87) achieved ‘*Very High*’ or ‘*High*’ level of agreement. The additional item included in Round 2 achieved a ‘Very High’ level of agreement. Of the seven items which were re-worded following Round 1, three items increased in level of agreement. Nine items in Round 1 achieved ‘*Very High*’ agreement; this increased to 12 in Round 2. In terms of response shift, the level of agreement remained stable for the majority of items on the questionnaire, however:

- 18 items increased level of agreement.
- 6 items decreased level of agreement.

### Stage 3: International consensus agreement

#### Participation

Seventeen participants took part in the Nominal Group discussions, with 15 taking part in the vote. The expert panel included Delphi participants and volunteer coordinators from the iLIVE Project Volunteers work package. The panel also included three members of the project research team (JE/SM/TM) who facilitated the discussion and voting. The panel constituted a multi-professional group, and included the following areas of expertise: Medicine; Nursing; Social Research (Psychologists, Sociologists and Humanistic Studies); Educational Psychology; Health Economics; Volunteer Service Management.

#### Nominal group technique discussion and consensus

**Figure 2.**
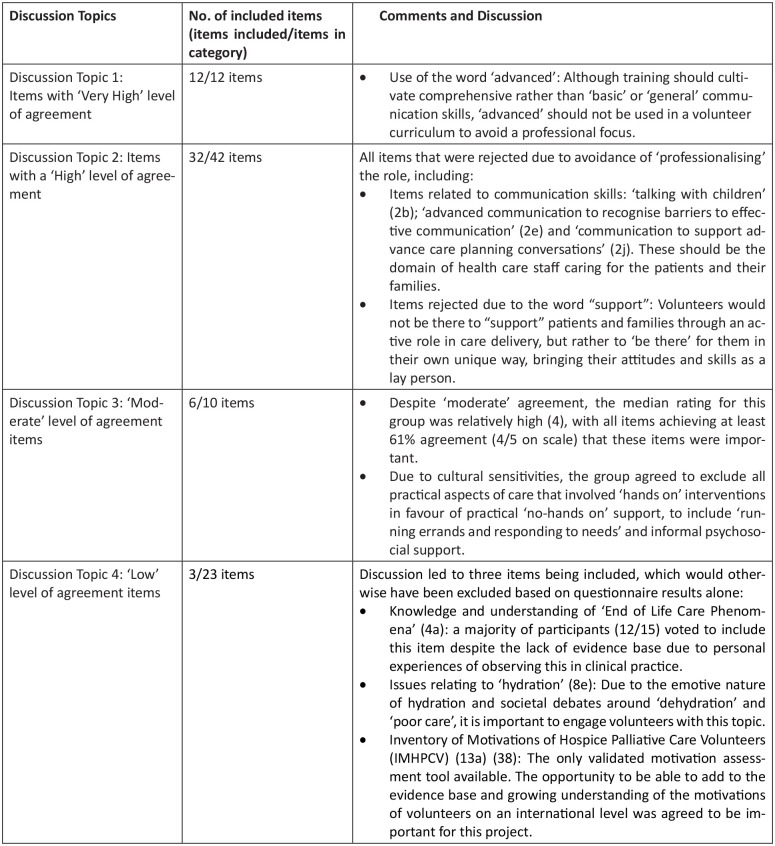
Nominal Group discussion and consensus.

Figure 2 below provides a summary of the discussion and comments from the Nominal Group and outcome of the voting. Table 4 displays the final list of 53 included items. Nine items were included following a majority vote, with each item receiving between 10 and 14 votes out of a total of 15 participants. Items included following a majority vote are highlighted in Table 4 with an asterisk (*).

**Table 4.** Final list of 53 included items, following the outcome of the Nominal Group meeting.

Included items	
Section 1: ‘being there’ and ‘being present’ with the patient/family	
This category relates to the concept of ‘active relational skills’, that is, what characterises a ‘good relationship’ between patients and volunteers. For example to ‘be there’ for someone takes unconditional acceptance, empathy, authenticity, warmth, understanding, sensitivity, honesty, involvement, respect, attention and enthusiasm. Training should enhance and hone these skills and qualities.	
1a	Establishing an environment of ‘mutuality’ to promote empathy and a ‘non-judgemental’ relationship
1b	Being attentive to the emotional needs of persons at the end of life (e.g. listening to the patient’s/family’s fears, worries, hopes, dreams, other feelings, etc.)
1c	Being responsive to the ‘uniqueness of the other’
1d	How to be ‘present’ with patients and families
1e	Relational attunement; establishing a connection, building rapport and relationship building with patients and families
1f	Journeying with patients, sitting with patients in the last hours of life
1g	Providing social support to patients and their families (e.g. talking with patients/families, sharing hobbies and interests, reading to the patient, etc.)
1h	Understanding the social nature of the volunteer role
1i	Use of humour in patient/volunteer interactions
Section 2: communication skills	
This category relates to the ‘Instrumental’ elements which can underpin good communication skills, for example ‘learned’ communication skills that adhere to a ‘formal’ learning and teaching agenda. These are ‘taught skills’ which provide volunteer with a ‘framework’ to guide their communication and engagement with patients and families.	
2a	Communication skills training: listening skills and responding to patient and family emotions
2d*	Communication with patients with dementia and cognitive decline
2g*	Understanding issues of collusion, including when and how to address this with the patient’s care team
2h	General Introduction to Communication Skills: the need for good communication skills
Section 3: cultural competency	
Cultural competence can be defined as the ability to understand, communicate with and effectively interact with people with diverse cultures, values, beliefs and feelings. Cultural competence encompasses being aware of one’s own world view, developing positive attitudes towards cultural differences, gaining knowledge of different cultural practices and world views.	
3a	Understanding diversity and seeing patients and their families as individuals
3b	Understanding personal values, belief systems, attitudes, judgements and worldviews and how these may impact on the care and support provided to patients and their families
3d	Peer Support: Activities/resources to build and facilitate strong relationships with other volunteer colleagues, to discuss difficult situations or patients, to ask questions and give or receive advice in a friendly non-judgemental environment
3e	Understanding behaviours related to fears around death and dying, including ‘fear of death’ and ‘death anxiety’
Section 4: end of life phenomena	
End-of-life phenomena has been defined by Claxton-Oldfield^39^ as ‘unusual happenings that occur shortly before, at the time of or shortly after a person dies’	
4a*	Knowledge and understanding of different End-of-life phenomena
Section 5: defining and promoting understanding of the volunteer role	
This category relates to defining the role of the volunteer, in the care of patients in the last hours of life and their families. This refers to establishing definitions of role, practice and the volunteer ‘place’ within the organisation.	
5a	Understanding the ‘definition’ of the volunteer role within the service
5b	Understanding the volunteer role as part of the care team
5c	Understanding of the complexities of the care environment and the role of the volunteer within it; exploring power relationships between volunteer/staff and volunteer/patient and family
Section 6: ethical issues relating to end of life care and the volunteer role	
This category has been included to highlight the complexity of the volunteer role and the relationships that are built with patients, families and explore the potential ethical conflicts this could generate.	
6a	Issues of confidentiality and how to navigate this within the volunteer role
6b	How to ensure confidentiality is upheld whilst undertaking the volunteer role
6c	Negotiating ‘boundary spaces’ within the role of a volunteer (e.g. not ‘friend’ or ‘professional’ and not ‘paid’ member of the organisation)
6d	Understanding of ethical Issues that could be encountered as part of the volunteer role (e.g. ethical dilemmas, competing interests, receiving gifts, clinical concerns, etc.)
6e	Understanding ethical issues in palliative and end of life care (e.g. assisted suicide, hastening death, etc.)
6f	Dealing with experiences of ‘powerlessness’ within the volunteer role, avoiding burnout and promoting resilience (e.g. often the volunteer role is to ‘be there’ with patients and families rather than actively ‘doing’ for them, leaving the potential to feel ‘powerless’ and frustrated with help they can offer)
Section 7: loss, grief and bereavement	
This category reflects the emotional impact of life-threatening illness and end of life on patients and families. Understanding loss and the diverse ways that people respond to loss may be pertinent for volunteers caring for patients at the end of life in the hospital setting.	
7a–c	Understanding processes of loss, grief and bereavement
7f	Exploring personal experiences of grief and how this may impact the volunteer in their role
Section 8: physical signs and symptoms in palliative and end of life care	
This category reflects findings in the literature that suggest a basic knowledge of the common symptoms associated with life-limiting conditions, and signs and symptoms of approaching death, as potentially useful in reducing anxiety, whether their own or that of patients or families.	
8a	Prepare the volunteer for naturally occurring changes in the patient towards the end of life, including how to communicate this to family members
8c	Understanding common symptoms at the end of life
8e*	Understanding of issues of hydration at the end of life
8j	Caring for ‘actively dying’ patients (e.g. days/hours leading up to death)
Section 9: practical aspects of the volunteer role (delivering care and support)	
Literature from this review highlighted a range of different ‘practical’ aspects of the volunteer role. While some volunteer services advocated for ‘hands on’ and ‘direct’ care from volunteers such as massage/touch, other services preferred volunteers to be involved in less direct care such as ‘running errands’.	
9c	Establishing a process of ‘handover’ between volunteers to support continuity of care
9d	Providing practical support to patients and their families (e.g. running errands and responding to needs)
Section 10: psychological/psychosocial aspects of care at the end of life	
This category reflects that for some volunteer services, psychosocial and existential elements of care have been highlighted as a core part of ‘tasks’ undertaken by volunteers. Ensuring volunteers are equipped to engage in this aspect of care necessitates increased volunteer training provision.	
	*No items were included from this section*
Section 11: religion and spirituality	
11a	Understanding the difference between religious and spiritual needs
11b	Understanding and acceptance of, and respect for, the spiritual needs of persons at the end of life
11c	Understanding spiritual diversity
11d	Being aware of religious/spiritual needs of patients and their families, and being able to ‘signpost’ for further support if required
Section 12: volunteer as patient/family advocate	
In some instances, volunteers can occupy a ‘middle ground’ between paid health-care professionals (eg, doctors and nurses) and the patient’s family and friends. As such, volunteers occupy a space outside both professional and family roles. Volunteers may become aware of patient/family needs that are not being met, providing opportunity to advocate for those patients, or support families to advocate for themselves.	
12b	Understanding patient rights
Training programme infrastructure: issues of responsibility to the volunteer and the care providing organisation The following categories highlight organisational issues related to setting up a volunteer service and embedding it into the organisational structure.	
Section 13: volunteer recruitment/retention	
13a*	Use of ‘motivation’ (to be a volunteer) assessment tool as part of the volunteer selection process
Section 14: volunteer support	
14a	Self-care information and strategies and personal resilience
14b	Regular ongoing mentoring
14d*	Rituals in dying: practicing ‘rituals’ and other ways to honour the lives of patients
14e	Establish an environment for informal supervision/formal structured supervision with feedback
14f	Coping Strategies for dealing with suffering and death
14g*	Access to wider support services and Psychological support
14h	Training updates and other ongoing educational opportunities
Section 15: community engagement and advocacy for the volunteer programme	
15b*	Engaging with staff and management within the care providing organisation, to promote the work of the volunteer service
Section 16: volunteer competency and volunteer assessment	
16a*	Development of ‘Core Competencies’ for volunteers providing support to patients in the last days of life, and their families
16b	Development/agreement of ‘standard’ outcome measures to evaluate benefit of the programme
Section 17: issues of organisational infrastructure and implementation	
17a	Embed the volunteer service within the organisation, with attention to organisational/regional/national/international legislation affecting volunteers
17b	Establish organisational policy and procedures for role of the volunteer service and volunteer coordinator

* Items included following a majority vote during the Nominal Group Meeting.

## Discussion

### Main findings from the study

This Delphi process gained consensus on 53/87 items (61%) to be incorporated into the European Core Curriculum for hospital palliative care volunteers, for services that support patients in the last weeks of life (Table 4). The constructivist nature of the Delphi process was a key benefit for this study. The Nominal Group facilitated reflection and in-depth discussions on individual items, resulting in a more nuanced process than relying on quantitative analysis alone. Discussions also highlighted important issues to consider when thinking about how these items should be incorporated into a subsequent European Core Curriculum for end-of-life-care volunteer services.

### Strengths and limitations

The Delphi process was a useful method to enable consensus to be established. A key strength in the methodology for this study was in the use of Nominal Group Technique to confirm final consensus. The Nominal Group provided an opportunity for more nuanced discussion regarding individual items, gave participants time to discuss items in greater depth, which resulted in items being included that would have otherwise been excluded based on the quantitative results alone.

The scoping review within this study was conducted with the specific aim to generate items for inclusion on the Delphi questionnaire, rather than provide a narrative summary or meta-analysis of the papers identified. Therefore, the results of this element of the study must be interpreted alongside the Delphi process as a whole. Another limitation concerns the lack of volunteer perspectives represented in the Delphi process. The sampling process was designed to maximise participation using snowball sampling, however, there remains a predominance of healthcare professional perspectives. The Delphi questionnaire included six volunteer managers/coordinators in Round 1 and Round 2, with four palliative care volunteers in Round 1 and three in Round 2, however they were not asked the setting in which they volunteer.

### What this study adds?

This Delphi process underlined the perspective that volunteers occupy a unique space, bridging the gap between the clinical environment and patients and relatives.^40,41^ Items that focussed on the ‘relational’ aspects of the end-of-life-care volunteer role were given priority within the Nominal Group discussions, specifically to distinguish them from clinical (and wider health care professional) roles. Literature from the scoping review revealed that establishing boundaries was important to promote good working relationships between volunteers and staff, especially in more ‘structured’ settings^9^ such as a hospital. Findings from this study reinforce that defining and setting boundaries should be a key element for planning and developing hospital end-of-life-care volunteer services to ensure clarity regarding role and responsibility, as well as developing appropriate volunteer training to help volunteers navigate their specific roles and activities within the unique space of the hospital.^9^ The fact that volunteers are not ‘professionalised’ and that they represent the ‘community’ around them, is part of their unique benefit,^41,42^ however, ensuring their role does not cross over into more formal ‘advocacy’ roles has been identified as an important clarification.^4,43,44^ Establishing clear parameters of working, including how volunteers work alongside paid staff, can mitigate the potentially stressful nature of the role.^4^ This Delphi recommends that hospital end-of-life-care volunteers should be supported to develop informal roles of support and presence, ensuring clear role boundaries to distinguish them from clinical support. The developed European Core Curriculum includes specific attention to ensuring end-of-life-care volunteers are embedded within the organisation, including understanding the specific needs of wards within the hospital where the volunteers will be supporting dying patients.

Volunteers can be a conduit for information between clinical staff and patients/relatives, providing peace of mind at a challenging time,^40^ particularly in the hospital setting if family or friends are unable to be with the patient. For some relatives, having someone to be there if they cannot may be a welcome compromise that could mitigate feelings of guilt resulting from leaving loved ones alone.^12,13^ Indeed, items reflecting ‘being there’ and ‘being present’ rated highly in the Delphi questionnaire, confirming these should be key skills addressed within the volunteer training programme.^16^ Research suggests that the social or relational nature of the role is a core theme within palliative care volunteer narratives,^17^ as well as being a motivator for becoming a volunteer.^45^ Volunteers have also reported that their roles have been enriched by the insights they have gained into the lives of patients through their interactions.^20^ However, training of end-of-life-care volunteers in the hospital must acknowledge the specific challenges to providing this type of relational support to dying patients in this setting, and support volunteers appropriately. For example, constantly changing acute care environments and ward settings as well as high patient turnover all limit the potential to build relationships with patients as well as restricting continuity of support.^18^ For volunteers supporting dying patients in the hospital setting, the ability to establish a connection may be further compromised due to the deteriorating condition of the patient, as well as exposing the volunteer to potentially difficult and challenging situations as the death of a patient approaches. Ensuring that any developed service and training programme is attentive to these specific challenges has the potential to maintain volunteer motivation, sense of satisfaction and retention of volunteers in the service.

Literature shows that some volunteer services advocate for ‘hands on’ and ‘direct’ care such as massage/touch,^46^ however, other evidence suggests this may not always be appropriate and that less ‘hands on’ care may be more important to patients^47^ or acceptable to staff.^18^ Consensus from the Delphi was to exclude elements of service provision and volunteer training related to all aspects of care that involved ‘hands on’ interventions and to include more practical ‘no-hands on’ support such as ‘running errands’, ‘responding to needs’ and informal psychosocial support. Discussion in the Nominal Group suggested engaging in ‘hands on’ care with patients who are entering the last weeks or days of life may be too culturally sensitive to be included as a standardised core component for end-of-life-care volunteers in a European Core Curriculum. Although studies have described a wide range of hands on activities engaged in by palliative care volunteers in the hospital setting, there remains a certain reticence over whether it is appropriate for volunteers to undertake such tasks.^18^ Reflecting this cautious view, the recommendations from this study suggest that this element of service provision for end-of-life-care volunteers, including specific training needed as a result, should instead be locally defined as part of the adaptation of the curriculum within individual countries or organisations.

End-of-life-care volunteers will likely be very different to existing volunteers in the hospital, both in terms of the activities they may undertake and the qualities and skills they will require.^18^ They may be more focussed on learning how to ‘be present’, with a core focus on the relational aspects of individual encounters, rather than more task oriented volunteering that may be in place in the wider organisation.^16,17,40^ Developing core competencies for end-of-life-care volunteers in the hospital setting, it was agreed, would underpin the ethos and values of the service as separate to generic volunteers in the wider hospital, and which should guide the delivery of volunteer training. This must also include clear role descriptions specific to hospital end-of-life-care volunteers.^18^ Ensuring that volunteers have clearly defined roles and responsibilities, including clear lines of support, has been highlighted as important to ensure clear understanding of the aims of the volunteer service and promote their place within the wider healthcare team,^18^ and this will be especially pertinent for any developed end-of-life-care volunteer service.

## Conclusion and implications for practice

The developed European Core Curriculum created following this Delphi study is a potentially useful tool to underpin the training of volunteers, and the implementation of end-of-life-care volunteer services within the hospital setting. As well as identifying key topics for volunteer training, findings from the Delphi study highlighted that any curriculum must include steps for embedding the end-of-life-care volunteer service within the organisational infrastructure. This was seen as important to ensure that the service is recognised as a core part of the care provided to dying patients within the organisation, with established roles and responsibilities, as well as ensuring volunteers as part of the service have access to hierarchies of support. This was seen as particularly relevant for end-of-life-care volunteers within the hospital, and reflects recent literature highlighting the unique challenges associated with this care setting.^18^ Recommendations for further research include further validation of this core curriculum with key stakeholders including volunteers, in-depth examination of the barriers and facilitators to the implementation of end-of-life-care volunteer services and volunteer training in hospitals, alongside research into the experiences of key stakeholders. For example, end-of-life-care volunteers, volunteer coordinators and hospital staff experiences of providing and facilitating volunteer support to dying patients, and most importantly, including the patient voice to understand how the volunteer service has affected their experience of care.

## Supplemental Material

sj-pdf-1-pmj-10.1177_02692163211045305 – Supplemental material for ILIVE Project Volunteer study. Developing international consensus for a European Core Curriculum for hospital end-of-life-care volunteer services, to train volunteers to support patients in the last weeks of life: A Delphi study Click here for additional data file. Supplemental material, sj-pdf-1-pmj-10.1177_02692163211045305 for ILIVE Project Volunteer study. Developing international consensus for a European Core Curriculum for hospital end-of-life-care volunteer services, to train volunteers to support patients in the last weeks of life: A Delphi study by Tamsin McGlinchey, Stephen R Mason, Ruthmarijke Smeding, Anne Goosensen, Inmaculada Ruiz-Torreras, Dagny Faksvåg Haugen, Miša Bakan and John E Ellershaw in Palliative Medicine

sj-pdf-2-pmj-10.1177_02692163211045305 – Supplemental material for ILIVE Project Volunteer study. Developing international consensus for a European Core Curriculum for hospital end-of-life-care volunteer services, to train volunteers to support patients in the last weeks of life: A Delphi study Click here for additional data file. Supplemental material, sj-pdf-2-pmj-10.1177_02692163211045305 for ILIVE Project Volunteer study. Developing international consensus for a European Core Curriculum for hospital end-of-life-care volunteer services, to train volunteers to support patients in the last weeks of life: A Delphi study by Tamsin McGlinchey, Stephen R Mason, Ruthmarijke Smeding, Anne Goosensen, Inmaculada Ruiz-Torreras, Dagny Faksvåg Haugen, Miša Bakan and John E Ellershaw in Palliative Medicine

sj-pdf-3-pmj-10.1177_02692163211045305 – Supplemental material for ILIVE Project Volunteer study. Developing international consensus for a European Core Curriculum for hospital end-of-life-care volunteer services, to train volunteers to support patients in the last weeks of life: A Delphi study Click here for additional data file. Supplemental material, sj-pdf-3-pmj-10.1177_02692163211045305 for ILIVE Project Volunteer study. Developing international consensus for a European Core Curriculum for hospital end-of-life-care volunteer services, to train volunteers to support patients in the last weeks of life: A Delphi study by Tamsin McGlinchey, Stephen R Mason, Ruthmarijke Smeding, Anne Goosensen, Inmaculada Ruiz-Torreras, Dagny Faksvåg Haugen, Miša Bakan and John E Ellershaw in Palliative Medicine

sj-pdf-4-pmj-10.1177_02692163211045305 – Supplemental material for ILIVE Project Volunteer study. Developing international consensus for a European Core Curriculum for hospital end-of-life-care volunteer services, to train volunteers to support patients in the last weeks of life: A Delphi study Click here for additional data file. Supplemental material, sj-pdf-4-pmj-10.1177_02692163211045305 for ILIVE Project Volunteer study. Developing international consensus for a European Core Curriculum for hospital end-of-life-care volunteer services, to train volunteers to support patients in the last weeks of life: A Delphi study by Tamsin McGlinchey, Stephen R Mason, Ruthmarijke Smeding, Anne Goosensen, Inmaculada Ruiz-Torreras, Dagny Faksvåg Haugen, Miša Bakan and John E Ellershaw in Palliative Medicine
