# Supplementary material for: ILIVE Project Volunteer study. Developing international consensus for a European Core Curriculum for hospital end-of-life-care volunteer services, to train volunteers to support patients in the last weeks of life: A Delphi study
Source: Palliat Med. 2021 Oct 20;36(4):652–70. doi: 10.1177/02692163211045305 (PMC9006393; doi:10.1177/02692163211045305)
Supplement: sj-pdf-1-pmj-10.1177_02692163211045305 – Supplemental material for ILIVE Project Volunteer study. Developing international consensus for a European Core Curriculum for hospital end-of-life-care volunteer services, to train volunteers to support patients in the last weeks of life: A Delphi study [file sj-pdf-1-pmj-10.1177_02692163211045305.pdf]

Table of Included Studies: iLIVE Volunteer Study: Scoping Review to Inform Development of Delphi Questionnaire

	Article Title	Reference	Authors	Key Words	Abstract	KEY:<br>**Bold Caps: Categories**<br>**Bold lower case: Sub categories/Items**
1	Understanding the role of the volunteer in specialist palliative care: a systematic review and thematic synthesis of qualitative studies.	BMC Palliative Care. 13(1):3, 2014 Feb 10.	Burbeck R<br><br>Candy B<br><br>Low J<br><br>Rees R		BACKGROUND: Volunteers make a major contribution to palliative patient care, and qualitative studies have been undertaken to explore their involvement. With the aim of making connections between existing studies to derive enhanced meanings, we undertook a systematic review of these qualitative studies including synthesising the findings. We sought to uncover how the role of volunteers with direct contact with patients in specialist palliative care is understood by volunteers, patients, their families, and staff.<br><br>METHODS: We searched for relevant literature that explored the role of the volunteer including electronic citation databases and reference lists of included studies, and also undertook hand searches of selected journals to find studies which met inclusion criteria. We quality appraised included studies, and synthesised study findings using a novel synthesis method, thematic synthesis.<br><br>RESULTS: We found 12 relevant studies undertaken in both inpatient and home-care settings, with volunteers, volunteer coordinators, patients and families. Studies explored the role of general volunteers as opposed to those offering any professional skills. Three theme clusters were found: the distinctness of the volunteer role, the characteristics of the role, and the volunteer experience of the role. The first answers the	**VOLUNTEER ROLE**<br>**Volunteer Role: Task Vs Presence**<br>Understanding the social nature of the role/forming relationships.<br><br>**Establishing a definition of the volunteer role within the service:** Establishing parameters of working can reduce stressful nature of the role: addressing role ambiguity and how volunteers work alongside other paid staff.<br><br>**Understanding power relationships:** Volunteer/patient and volunteer/staff relationships. Including how staff ‘control’ volunteers.<br><br>**VOLUNTEER SUPPORT**<br>**Volunteer Support**: regular ongoing mentoring.<br><br>**ORGANISATIONAL/IMPLEMENTATION**<br>**Power relations**: Volunteers can see their role as subordinate to that of paid staff.<br><br>**Legislation affecting volunteers** Regional, National, International. Considerations about how these impact on the role of volunteers.

					question, is there a separate volunteer role? We found that to some extent the role was distinctive. The volunteer may act as a mediator between the patient and the staff. However, we also found some contradictions. Volunteers may take on temporary surrogate family-type relationship roles. They may also take on some of the characteristics of a paid professional. The second cluster helps to describe the essence of the role. Here, we found that the dominant feature was that the role is social in nature. The third helps to explain aspects of the role from the point of view of volunteers themselves. It highlighted that the role is seen by volunteers as flexible, informal and sometimes peripheral. These characteristics some volunteers find stressful.<br><br>CONCLUSIONS: This paper demonstrates how qualitative research can be sythnesised systematically, extending methodological techniques to help answer difficult research questions. It provides information that may help managers and service planners to support volunteers appropriately.	
2	Training programs in communication skills for health care professionals and volunteers.	Indian Journal of Palliative Care. 17(Suppl):S12-3, 2011 Jan.	Rajashree K	Communication skills; Palliative care; Training programmes	Communication skills are as important as vital needs. Health care professionals have to be aware of their own communication practices and need to undergo periodic appraisal of the same. Training programmes in communication skills are unfortunately not part of our academic curriculum. The article highlights the need and the overview of such training programmes.	**VOLUTEER TRAINING PROGRAMME:**<br>• 16 hour training programme<br>• Advanced: 100 hour programme<br><br>**COMMUNICATION SKILLS**<br>**General Introduction to CS:** the need for good communication skills<br><br>**Use of clinical scenarios:** identify the patient as a person with a disease rather than a "case'<br><br>**Listening techniques:** enhancing interaction between the patient and the volunteer

						**Barriers to communication:** For example 'distancing behaviour'<br>**DELIVERY OF TRAINING**<br>**Simulated experience: Role Plays, group work, facilitated discussions**
3	Implementing the 'No One Dies Alone program': process and lessons learned.	Geriatric Nursing. 35(6):471-3, 2014 Nov-Dec.	Bradas C<br><br>Bowden V<br><br>Moldaver B<br><br>Mion LC			**VOLUTEER TRAINING PROGRAMME:**<br>• **4 hour workshop**<br>(4-h block held on a Saturday rather than spread out after work)<br><br>**PHYSICAL SIGNS AND SYMPTOMS**<br>**Recognising Changes:** changes in a patient's condition that are indications of impending death.<br><br>**Isolation precautions** (no description of what this is)<br><br>**VOLUNTEER SUPPORT**<br>**Self-care information and strategies**.<br>**Volunteer Support**: overcoming the 'stressors' of the volunteering role.<br><br>**VOLUNTEER ROLE**<br>**Comfort measures and strategies to support the patient**: CD player, a variety of genres of CDs,<br><br>**Process of 'handover' between volunteers:** diary to document the present 'vigil' for the oncoming volunteer,<br><br>**ORGANISATIONAL/IMPLEMENTATION**<br>**Certification following successful completion of training:** volunteers receive a certificate and badge designating them as Compassionate Companions in the NODA Program.

4	Volunteers in specialist palliative care: a survey of adult services in the United Kingdom.	Journal of Palliative Medicine. 17(5):568-74, 2014 May.	Burbeck R<br><br>Low J<br><br>Sampson EL<br><br>Bravery R<br><br>Hill M<br><br>Morris S<br><br>Ockenden N<br><br>Payne S<br><br>Candy B		BACKGROUND: Worldwide, the demand for specialist palliative care is increasing but funding is limited. The role of volunteers is underresearched, although their contribution reduces costs significantly. Understanding what volunteers do is vital to ensure services develop appropriately to meet the challenges faced by providers of palliative care.<br><br>OBJECTIVE: The study's objective is to describe current involvement of volunteers with direct patient/family contact in U.K. specialist palliative care.<br><br>DESIGN: An online survey was sent to 290 U.K. adult hospices and specialist palliative care services involving volunteers covering service characteristics, involvement and numbers of volunteers, settings in which they are involved, extent of involvement in care services, specific activities undertaken in each setting, and use of professional skills.<br><br>RESULTS: The survey had a 67% response rate. Volunteers were most commonly involved in day care and bereavement services. They entirely ran some complementary therapy, beauty therapy/hairdressing, and pastoral/faith-based care services, and were involved in a wide range of activities, including sitting with dying patients.<br><br>CONCLUSIONS: This comprehensive survey of volunteer activity in U.K. specialist palliative care provides an up-to-date picture of volunteer involvement in direct contact with patients and their families, such as providing emotional care, and the extent of their	**VOLUNTEER ROLE (Common activities)**<br>**Emotional Care and Support:** how to support patients and their families<br><br>**Sitting with patients:** patients in the last hours of life.<br><br>**VOLUNTEER SUPPORT**<br>**Self-care information and strategies**.<br>**Volunteer Support**<br>This paper highlights the emotionally demanding role of the volunteer in end of life care: emotionally challenging activities that 'paid' staff may not be funded to do and demonstrates how much volunteers contribute to core end-of-life care.

					involvement in day and bereavement services. Further research could focus on exploring their involvement in bereavement care.	
5	Role ambiguity, role conflict, or burnout: are these areas of concern for Australian palliative care volunteers? Pilot study results.	American Journal of Hospice & Palliative Medicine. 31(7):749-55, 2014 Nov.	Phillips J<br><br>Andrews L<br><br>Hickman L	burnout; interdisciplinary team; palliative care; role ambiguity; role conflict; volunteer	OBJECTIVE: To determine whether burnout, role ambiguity, or conflict affects Australian hospice volunteers.<br><br>METHOD: Hospice volunteers (n = 120) were invited to participate in this pilot survey. Quantitative data were analyzed using descriptive statistics, while the free-text responses were analyzed using thematic content analysis. The Strengthening the Reporting of Observational Studies in Epidemiology (STROBE) Guidelines have been used to report this data.<br><br>RESULTS: A total of 97 participants completed the survey. The majority were middle-aged women who had been palliative care volunteers for more than 7 years and volunteered 14 hours/week (median). Participants reported low levels of role ambiguity (x = 8.4, standard deviation [SD] +/-3.0) and conflict (x = 9.8, SD +/-3.4) and described enjoying their volunteering and having no symptoms of burnout (76%).<br><br>SIGNIFICANCE: Active hospice volunteers report low levels of role ambiguity, conflict, and burnout. Adopting a range of self-care strategies and working within a structured volunteer program appear to be important protective factors.<br>Copyright © The Author(s) 2013.	**VOLUNTEER ROLE**<br>**Establishing a 'definition' of the volunteer role within the service:** Operating within a structured volunteer program helps minimize these risks by ensuring that volunteers are adequately prepared and supported in their roles<br><br>**Understanding of the complexity of the hospice/end of life care environment**<br><br>**VOLUNTEER SUPPORT**<br>**Self-care information and strategies**.<br>**Volunteer Support**: regular ongoing mentoring.

6	Promoting volunteer capacity in hospice palliative care: a narrative review. [Review]	American Journal of Hospice & Palliative Medicine. 31(1):69-78, 2014 Feb.	Pesut B<br><br>Hooper B<br><br>Lehbauer S<br><br>Dalhuisen M	end of life; hospice; palliative; review; terminal; volunteers	Hospice volunteers play an essential role in the primary care network for end of life. The purpose of this review was to examine the evidence on hospice volunteers published between 2002 and July 2012. An electronic search of PubMed, CINAHL and PsychINFO using controlled vocabulary, and a reference scan, yielded 54 studies focusing on hospice volunteers. Studies were primarily descriptive using quantitative, qualitative and mixed methods. Findings from studies were grouped thematically into descriptions of the work of hospice volunteers; recruitment, preparation and retention of hospice volunteers; and perspectives and outcomes of the volunteer role. A substantial body of evidence exists describing the roles, stresses and rewards of hospice volunteering. Less is known about how to adequately recruit, prepare and retain volunteers. A small but intriguing body of evidence exists around volunteers' contributions to family satisfaction and patient longevity. Although the evidence around hospice volunteers continues to grow, there is an urgent need for further research. Findings indicate that volunteers make important contributions to high quality end of life care. However, more focused research attention is required to better understand how to maximize this contribution while providing better support for volunteers.	**LEADERSHIP**<br>**how to better support hospice volunteers**<br><br>**VOLUNTEER ROLE**<br>**Volunteer Role: Task Vs 'Presence'**<br>Understanding the social nature of the role/forming relationships.<br><br>**The volunteer as patient advocate**<br><br>**Emotional Care and Support:** how to support patients and their families<br><br>**Sitting with patients:** patients in the last hours of life.<br><br>**DEATH ANXIETY**<br><br>**CULTURAL COMPETENCY**<br>**how to support patients with diverse values, beliefs, and feelings**<br><br>**COMMUNICATION SKILLS**<br><br>**ETHICAL ISSUES**<br>**Ethical issues in the volunteer role:** For example, what to do when patients/families offer gifts, being asked to do things outside of their role, seeing less than optimal care, hearing patients talk of suicide.<br><br>**VOLUNTEER SUPPORT**<br>**Self-care information and strategies**.<br>**Volunteer Support**: overcoming the ‘stressors’ of the volunteering role.<br><br>**ORGANISATIONAL/IMPLEMENTATION**

						**Power relations**: Volunteers can see their role as subordinate to that of paid staff.
7	Last watch: developing an inpatient palliative volunteer program for U.s. Veterans in hospice.	Omega - Journal of Death & Dying. 67(1-2):87-95, 2013.	Gerber PS		This project offers encouraging evidence of the impact of providing companionship and compassionate care to actively dying Veterans. Veteran-centered care serves as a guide for volunteer program objectives including goals based on measuring outcomes of volunteer visits offered to Veterans, frequency of visits to those desiring volunteer involvement and documentation of goals on the resident plan of care. Forty-eight Veterans were admitted to the Palliative Integrated Care (PIC) Unit during the project review period with a median length of stay (LOS) of 7 days, ranging 1-50 days. Goals were met for Veterans with LOS > 3 days; however, it was more challenging to meet the needs of Veterans with shorter stays. Data for this project were collected over a 6-month period. The U.S. Department of Veterans Affairs (VA) is responding to the needs of end-of-life Veterans who do not have family or friends readily available through the No Veteran Dies Alone initiative.	**VOLUTEER TRAINING PROGRAMME (veterans hospice):**<br>• independent study program; volunteers learn at own pace<br>• offered two options to suit their learning style:<br>○ Written manual<br>○ DVDs with identical information provided in an audiovisual format.<br><br>**ORGANISATIONAL ORIENTATION**<br>Overview of the 'mission and history' of the care environment.<br><br>**VOLUNTEER ROLE**<br>**confidentiality,**<br>**care and comfort**<br>**death and dying**<br>**grief and loss**<br>**patient rights**<br>**communication**<br>**spirituality and spiritual care**<br>**safety and infection control,**<br>**family**<br>**ethics**<br><br>**ASSESSMENT OF COMPETENCE**<br>**Formal Assessment:** essay questions to assess understanding, followed by face to face meeting with volunteer coordinator<br><br>**Ongoing assessment of 'competency':**<br>Volunteer competency was continually

						assessed through discussion and observation, input from the staff and the<br>Veterans, as well as regular review of the volunteer daily logs.<br><br>**TRAINING PROGRAMME EVALUATION**<br>Outcome Measures:<br>• Number of patients/volunteer episodes<br>• Percentage patients offered volunteer services >90%<br>• Minimum of one volunteer visit per week for 80% of patients<br>• 90% patients requesting volunteer visits would have volunteer goals on their plan of care
8	Personality traits of British hospice volunteers.	American Journal of Hospice & Palliative Medicine. 30(7):690-5, 2013 Nov.	Claxton-Oldfield S<br><br>Claxton-Oldfield J<br><br>Paulovic S	United Kingdom; big five; personality; recruitment; residential hospices; volunteers	In total, 120 British female hospice volunteers completed the NEO five-factor inventory (NEO-FFI) of Costa Jr and McCrae. The NEO-FFI measures the so-called big 5 personality traits of neuroticism, extraversion, openness, agreeableness, and conscientiousness. Compared to both American NEO-FFI norms for adult females and emerging British NEO-FFI norms for adult females, the hospice volunteers scored significantly lower, on average, in neuroticism and significantly higher, on average, in agreeableness and conscientiousness. No significant differences were found on any of the 5 traits between the British female hospice volunteers' scores and the NEO-FFI scores previously collected from a sample of Canadian female hospice palliative care volunteers. Implications for the recruitment of British hospice volunteers are discussed.	**VOLUNTEER SKILLS**<br>**Whole hearted commitment, compassion, level headedness combined with a non-judgemental attitude, and dedication**<br><br>**ORGANISATIONAL/IMPLEMENTATION**<br>**MOTIVATION TO BE A VOLUNTEER**<br>**Use of motivation assessment tool as a selection criteria**<br><br>***Main finding from study:***<br>Volunteer coordinators might consider using a personality assessment tool as part of the selection process. Some of the characteristics that are considered<br>important in volunteers (eg, respect for confidentiality, listening skills) can be fostered during training; others (e.g., Compassion, caring) are qualities that hospice programs cannot give people<br>‘‘to make them into good volunteers.’’

9	A study of the motivations of British hospice volunteers.	American Journal of Hospice & Palliative Medicine. 30(6):579-86, 2013 Sep.	Claxton-Oldfield S<br><br>Claxton-Oldfield J<br><br>Paulovic S<br><br>Wasylkiw L	United Kingdom; motivations; recruitment; residential hospices; retention; roles; volunteers	In all, 162 British hospice volunteers completed the Inventory of Motivations for Hospice Palliative Care Volunteerism (IMHPCV) of Claxton-Oldfield, Wasylkiw, Mark, and Claxton-Oldfield.(1) The IMHPCV taps into 5 different categories of motives for becoming a hospice palliative care volunteer: altruism, civic responsibility, leisure, self-promotion, and personal gain. Altruistic motives were the most influential reasons for choosing to join hospice; personal gain motives were the least influential reasons for becoming a hospice volunteer. Altruistic motives were found to be a significant predictor of volunteers' length of service to the hospice. Compared to previously collected data from a sample of Canadian hospice palliative care volunteers,(1) the current study's sample of British hospice volunteers scored significantly different on 2 of the 5 categories of motives on the IMHPCV.	**ORGANISATIONAL/IMPLEMENTATION**<br>**MOTIVATION TO BE A VOLUNTEER**<br>**Use of motivation assessment tool as a selection criteria**<br>A possible key to volunteer retention may be selecting prospective volunteers whose most important motivations for joining hospice are to help those in need; such volunteers may be more likely to remain active in the program longer.<br><br>**Outcome Measure:**<br>The authors encourage other researchers to use the IMHPCV as a tool for recruitment and retention.
10	A narrative literature review of the contribution of volunteers in end-of-life care services. [Review]	Palliative Medicine. 27(5):428-36, 2013 May.	Morris S<br><br>Wilmot A<br><br>Hill M<br><br>Ockenden N<br><br>Payne S		BACKGROUND: Volunteers are integral to the history of hospices and continue to play a vital role. However, economic, policy and demographic challenges in the twenty-first century raise questions about how best to manage this essential resource.<br><br>AIM: This narrative review explores the recent literature on end-of-life care volunteering and reflects upon the issues pertinent to current organisational challenges and opportunities.<br><br>DESIGN: The parameters of the review were set deliberately wide in order to capture some of the nuances of contemporary volunteer practices. Articles reporting on research or evaluation of adult end-of-life care services (excluding prison services) that use	**CULTURAL COMPETENCY:** need to address diversity issues in order to reach a larger ‘pool’ of potential volunteers and to support diverse clients.<br><br>**ETHICAL ISSUES**<br>**Ethical issues in the volunteer role:** negotiating the boundary spaces that volunteers inhabit, informality and regulation, ‘professional and friend’.<br><br>**ORGANISATIONAL/IMPLEMENTATION**<br>**Power relations**: Volunteers ‘place in the system.

					volunteers and were published in English between 2000 and 2011 were included.<br><br>DATA SOURCES: Seven electronic databases, key journals and grey literature databases.<br><br>RESULTS: Sixty-eight articles were included in the analysis. The articles were drawn from an international literature, while acknowledging that volunteer roles vary considerably by organisation and/or by country and over time. The majority of articles were small in scale and diverse in methodology, but the same topics repeatedly emerged from both the qualitative and quantitative data. The themes identified were individual volunteer factors (motivation, characteristics of volunteers, stress and coping, role boundaries and value) and organisational factors (recruiting for diversity, support and training and volunteers' place in the system).<br><br>CONCLUSIONS: The tensions involved in negotiating the boundary spaces that volunteers inhabit, informality and regulation, diversity issues and the cultural specificity of community models, are suggested as topics that merit further research and could contribute to the continuing development of the volunteer workforce.	

11	Training and supporting hospice volunteers: a regional survey.	American Journal of Hospice & Palliative Medicine. 29(5):355-61, 2012 Aug.	Lavenburg P<br><br>Bernt FM		We surveyed volunteers from 8 hospices in the Delaware Valley regarding training, perceived needs, and role satisfaction. Results were consistent with previous studies: satisfaction with preservice training and with volunteering was very high; respondents reported feeling very prepared and confident about doing hospice work as a result of their volunteer training. In addition, longer volunteer preservice training was associated with higher levels of overall satisfaction with training; levels of volunteer satisfaction and fulfillment tended to be lower during the first year of volunteering; and participation in volunteer support teams was associated with finding volunteer work rewarding and with feeling a part of the hospice team. Implications for preservice training and ongoing support and education of hospice volunteers are discussed.	**APPETITE FOR INTERNATIONAL CURRICULUM**<br><br>"some portion of a larger training requires orientation, completing paperwork, and so on; however, some large part of hospice training—for example, philosophy and history, principles of communication, pain management, bereavement, and so on— could arguably be interchangeable and shared, if training objectives, materials, and procedures were openly shared and discussed."
12	Improving cultural competency among hospice and palliative care volunteers: recommendations for social policy.	American Journal of Hospice & Palliative Medicine. 29(4):268-78, 2012 Jun.	Jovanovic M		This case study of 14 hospice and palliative care volunteers looked for recommendations and suggestions on how to increase cultural competency among hospice volunteers. In-depth interviews were conducted with a hospice in Toronto, Canada, and findings reveal that volunteers have very specific and diverse recommendations on how they prefer to be briefed and educated on cultural competency issues surrounding their patients. Findings also reveal hospice volunteers want more cultural competency training and acknowledge the importance of being culturally competent. This article concludes with a precis on recommendations for increasing cultural competency in hospice	**VOLUNTEER TRAINING**<br>**Ongoing learning around cultural competency**<br>**Reflexivity and awareness of own personal position**<br>**Understanding and respecting different cultural backgrounds and belief systems**<br>**Rituals in dying**<br><br>**VOLUNTEER SUPPORT**<br>**Learning from other volunteer experiences**<br>**Mentoring/bond building between volunteer community**<br><br>**DELIVERY OF TRAINING**<br>**Simulated experience: Role Plays, group work, facilitated discussions**

					and palliative care for both volunteers and agencies and discusses the top 4 future trends in cultural competency for hospice care.	
13	Cultural competency and diversity among hospice palliative care volunteers.	American Journal of Hospice & Palliative Medicine. 29(3):165-70, 2012 May.	Jovanovic M		This case study examines the current state of cultural competence in hospice and palliative care in the Greater Toronto Area (GTA). Because of changing demographic trends and ethnic minorities underutilizing hospice palliative care services, this research examined the current state of culturally competent care in a hospice setting, and the challenges to providing culturally competent care in a hospice in the GTA. A case study was conducted with a hospice and included in-depth interviews with 14 hospice volunteers. The findings reveal that volunteers encountered cultural clashes when their level of cultural competency was weak. Second, volunteers revealed there was a lack of adequate cultural competency training with	**VOLUNTEER TRAINING**<br>**Cultural Competency is important for training**<br>**Ongoing learning around cultural competency**<br>**Reflexivity and awareness of own personal position**<br>**Understanding and respecting different cultural backgrounds and belief systems**<br>**Communication skills**

					their hospice, and finally, there was a lack of ethnic, cultural, and linguistic diversity among the hospice volunteers.	
1<br>4	How they cope: a qualitative study of the coping skills of hospice volunteers.	American Journal of Hospice & Palliative Medicine. 28(6):398-402, 2011 Sep.	Brown MV		The purpose of this phenomenological study was to examine the coping techniques utilized by hospice volunteers. Individual semi-structured interviews were conducted with 15 hospice volunteers who had at least 1 year of experience, working as a hospice volunteer with direct patient care. The interviews were digitally recorded, transcribed, and analyzed utilizing qualitative research methods. The results indicated the volunteers used problem-focused coping (seeking advice from members of the hospice interdisciplinary team), emotion-focused coping (talking with others, going to funerals), meaning making through appraisal (religious beliefs, downward comparison), and physical techniques (walking, deep breathing). The most significant coping mechanism utilized for the volunteer was talking with the volunteer	Main Finding from Study:<br>When the participants were asked whether they had any suggestions to help deal with stress and improve coping, almost all of the participants stated that they had wonderful volunteer training.<br><br>**VOLUNTEER SUPPORT**<br>**Self-care information and strategies**.<br>**Volunteer Support**: regular ongoing mentoring.<br><br>**Organisational responsibility for ongoing psychological support**

					coordinator. Implications for hospice volunteer coordinators are also discussed.	
15	How can I help you? A study of the perceived importance of different kinds of hospice palliative care volunteer support.	American Journal of Hospice & Palliative Medicine. 28(4):271-5, 2011 Jun.	Claxton-Oldfield S<br><br>Gosselin N		A total of 143 adults were asked to imagine that they had recently been diagnosed with a life-threatening illness. After reading about the roles of hospice palliative care volunteers, participants were asked whether they would use the services of a volunteer to help them and their loved ones get through this difficult time. The vast majority (94.4%) of the participants said they would choose to have a volunteer. These participants were then asked to rate the importance of 23 different supportive tasks that volunteers can perform. These tasks reflected the different kinds of support--emotional, social, practical, informational, and religious/spiritual--that hospice palliative care volunteers typically provide. Overall, the practical support category (eg, "Having the volunteer run errands for me") received the highest mean importance rating. Significant gender differences were found for the categories of emotional support (eg, "Having the volunteer hold my hand") and social support (eg, "Having the volunteer share hobbies and interests with me"), with females rating both of these kinds of support as being more important to them than males did. The findings of this study may have practical implications for volunteer program coordinators.	**VOLUNTEER TRAINING**<br>Volunteer Training Programmes should include the following elements of training and support to encompass these activities:<br><br>**VOLUNTEER ROLE**<br>The major categories of support provided by direct patient/family care volunteers include<br>**1. Emotional support**<br>**2. Social support**<br>**3. Practical support**<br>**4. Informational support**<br>**5. Religious/spiritual support* (if this is what the patient/family wants)**<br>**6. Grief and bereavement support**<br><br>*This had the lowest mean 'importance' – however questions were limited to 'praying' and 'reading from scriptures' – other aspects of religious/spiritual support should be explored.

16	The stresses of hospice volunteer work.	American Journal of Hospice & Palliative Medicine. 28(3):188-92, 2011 May.	Brown MV		The purpose of this phenomenological study was to explore the interpretation of stress, the appraisal of the stressors, as well as the top stressors experienced by hospice volunteers. Individual semi structured interviews were conducted with 15 hospice volunteers. The interviews were digitally recorded, transcribed, and analyzed, using qualitative research methods. Although the results indicated that the hospice volunteers did not perceive their work as stressful, 2 main themes regarding challenging experiences did emerge. Hospice-related issues and personal issues were of concern to the volunteers. In addition, the timing of the stressors revealed that the most stress was felt at the beginning of their volunteer services, which has implications for hospice volunteer coordinators as they support their volunteers in the field.	Main idea from this paper: Little research exists that describes the stresses of hospice volunteers. Often stress can be 'hidden' – volunteers love their 'job' and altruistic motives mean they nay not describe their role as stressful despite elements causing some 'stress/distress'.<br><br>**DELIVERY OF TRAINING**<br>**Simulated experience: Role Plays, group work, facilitated discussions: "**Perhaps role-playing could demonstrate potential scenarios volunteers might come across and offer suggestions on how to handle those scenarios."<br><br>**VOLUNTEER SUPPORT**<br>**Self-care information and strategies**.<br>**Volunteer Support**: regular ongoing mentoring.<br>"Special attention should be given to new hospice volunteers as they tend to identify more stressful experiences during the first year of service."
17	It takes a whole community: the contribution of rural hospice volunteers to whole-person palliative care.	Journal of Palliative Care. 26(2):103-11, 2010.	McKee M<br><br>Kelley ML<br><br>Guirguis-Younger M<br><br>MacLean M<br><br>Nadin S		Although volunteers are widely acknowledged as important members of the palliative care team, their unique contribution to whole-person care has not been well documented or theorized, especially in rural communities. We conducted a focused ethnography in a small rural community, asking key community informants about their understanding of the role of hospice volunteers with dying people and their families. Our results show that these volunteers inhabit a unique third culture of care that fuses elements of formal care with the informal visiting of friends and neighbours. Their role is shaped to a community context	"Results are a reminder that it takes an entire community to care for the dying, and that hospice volunteers are a crucial link in the network of care that allows people to die with dignity and quality of life."<br><br>**VOLUNTEER ROLE**<br>**Link between the community and the patient**<br><br>**Emotional Care and Support:** how to support patients and their families

					where dying is not a private medical event, but rather a whole-person-in-community event, and where care is offered as a natural expression of the interdependence and reciprocity that characterizes rural community life. Our results are a reminder that it takes an entire community to care for the dying, and that hospice volunteers are a crucial link in the network of care that allows people to die with dignity and quality of life.	
18	Personality characteristics of hospice palliative care volunteers: the "big five" and empathy.	American Journal of Hospice & Palliative Medicine. 27(6):407-12, 2010 Sep.	Claxton-Oldfield S<br><br>Banzen Y		The goal of this study was to examine the personality characteristics of hospice palliative care volunteers by measuring the so-called big five personality traits and 4 separate aspects of empathy. A total of 99 hospice palliative care volunteers completed the NEO Five-Factor Inventory (NEO-FFI) of Costa Jr and McCrae and the Interpersonal Reactivity Index (IRI) of Davis. The vast majority (84%) of the volunteers were females. Compared to the norms for adult females on the NEO-FFI, female hospice palliative care volunteers scored significantly higher on the traits of agreeableness, extraversion, and openness and significantly lower on the trait of neuroticism. On the empathy measure, female hospice palliative care volunteers scored significantly higher on the empathic concern and perspective taking subscales compared to the female norms, and significantly lower on the personal distress and fantasy subscales. The results of this study may have implications for the recruitment and retention of hospice palliative care volunteers.	**ORGANISATIONAL/IMPLEMENTATION VOLUNTEER RECRUITMENT AND SELECTION**<br>**Use of motivation assessment tool as a selection criteria**<br><br>“Administering the NEO-FFI and IRI may help to reduce the costs (eg, time, effort, and money) that are associated with recruiting and training volunteers by screening out potentially unsuitable volunteers. There are, of course, other important considerations, besides personality characteristics, when it comes to volunteer selection, including a volunteer’s motives for volunteering, involvement in other volunteer activities, work and life experiences, and past experience with death and disease.”

1<br>9	Results from the national hospice volunteer training survey.	Journal of Palliative Medicine. 13(3):261-5, 2010 Mar.	Wittenberg-Lyles E<br><br>Schneider G<br><br>Oliver DP		BACKGROUND: Although the role of volunteers is at the heart of hospice care, little is known about hospice volunteer training and volunteer activity.<br><br>METHOD: A survey was used to assess current training programs for hospice volunteers. Hospices were invited to participate in the study from a link on the website for the Hospice Volunteer Association and Hospice Educators Affirming Life Project.<br><br>RESULTS: Survey results revealed that the majority of volunteer work is in patient care, with most hospice agencies requiring a minimum 12-month volunteer commitment and an average 4-hour volunteer shift per week. Volunteer training is separate from staff training, is provided by paid agency staff, and costs approximately $14,303 per year.<br><br>CONCLUSIONS: Communication and family support are considered important curriculum topics. Revisions to current volunteer training curriculum and format are suggested.	**VOLUNTEER ROLE:**<br>**VOLUNTEER TRAINING TOPICS**<br>“Consistent with findings in other national studies, the majority of volunteer activities remain central to patient care.”<br>**Training topic**<br><br>**Communication**<br>**Boundaries**<br>**Supporting families**<br>**Family dynamics**<br>**What is hospice care?**<br>**Policies and procedures**<br>**Being present**<br>**Grief and bereavement**<br>**Volunteerism and hospice**<br>**Spiritual care**<br>**Volunteer self-care**<br>**Cultural issues**<br>**Ethics and regulations**<br>**Care for actively dying**<br>**Physiology of dying**<br>**Diseases of dying**<br>**History of hospice**<br>**Practical bedside care**<br>**Rituals in dying**<br>**Volunteer monitoring**<br>**Touch/massage**<br>**Mediation**<br>**Music/art therapy**<br>**Pediatric care**<br><br>**DELIVERY OF TRAINING**<br>**Online Modules: “**online education can increase continuing education of volunteers, decrease educational costs, and potentially increase volunteer retention rates by providing specialized training consistent with the volunteer’s activity.”

20	How to attract more males to community-based hospice palliative care volunteer programs.	American Journal of Hospice & Palliative Medicine. 26(6):439-48, 2009 Dec-2010 Jan.	Claxton-Oldfield S<br><br>Guigne S<br><br>Claxton-Oldfield J		Two separate studies were conducted to better understand why so few middle-aged and older men volunteer in hospice palliative care; only about 10% of the patient/family care volunteers in New Brunswick's community-based hospice palliative care volunteer programs are men. In study 1, 15 (22%) of the 68 men who read a brief description about the kinds of things that hospice palliative care volunteers do expressed an interest in this type of volunteerism. The main reasons given for their lack of interest included "being too busy" and "not being able to handle it emotionally." At least one third of the men who said "No" to becoming a hospice palliative care volunteer expressed an interest in 10 of 13 other common volunteer activities (eg, driving). In study 2, 59 men were presented with a list of 25 tasks that hospice palliative care volunteers might perform when providing emotional, social, practical, and administrative support. The men were asked to indicate which tasks they would be willing to perform if they were a hospice palliative care volunteer. The men were least willing to serve on the board of directors (28%), provide hands on patient care (38%), and work in the volunteer program's office (42%); they were most willing to talk to the patient (97%), share hobbies and interests with the patient (92%), listen to the patient's memories and life stories (90%), and provide friendship and companionship (88%). The results of these studies may have implications for the recruitment of male volunteers to work with dying patients and their families.	**VOLUNTEER RETENTION**<br><br>**ORGANISATIONAL/IMPLEMENTATION**<br>**MOTIVATION TO BE A VOLUNTEER**<br>**Use of motivation assessment tool as a selection criteria**<br><br>**PERSONALITY CHARACTERISTICS OF HOSPICE PALLIATIVE CARE VOLUNTEERS**<br>**Adapting roles and responsibilities**<br><br>**HOSPITAL BASED VOLUNTEERS**<br>**Highly organised structure of hospitals impacts roles**<br>**Issues of role boundaries in clinical environment**<br>**Challenges of providing hands on care**<br>**Providing social and emotional support**<br>**Being there and being present**<br>"hospital based programs primarily provide social and emotional support to hospitalized patients, as well as opportunities for family members to take a break and know that someone will be there with their loved one. However, because they are visiting patients in the hospital, union work rules generally prevent volunteers from doing much in the way of hands on patient care (eg, lifting patients, turning patients in bed, helping with feeding)"

21	Motivations of hospice volunteers.	American Journal of Hospice & Palliative Medicine. 26(3):188-92, 2009 Jun-Jul.	Planalp S<br><br>Trost M		To recruit and retain volunteers, coordinators need to understand volunteers' motivations. In this study, 351 volunteers from 32 hospices in the western United States answered questions on a mailed survey about their motivations. The motivations reported were, in order of overall importance: to help others and learn, foster social relationships, feel better, and pursue career goals. Younger volunteers reported stronger career motivations, and retired and unemployed volunteers reported stronger social motivations. Volunteer coordinators should consider these motivations in communicating with potential and current volunteers, with special emphasis on compassion for those in need and the importance of helping, on fostering hospice volunteering as a learning experience, and in accessing and building social networks around hospice volunteering.	**VOLUNTEER RETENTION**<br><br>**ORGANISATIONAL/IMPLEMENTATION**<br>**MOTIVATION TO BE A VOLUNTEER**<br>**Use of motivation assessment tool as a selection criteria**<br><br>**VOLUNTEER ROLE**<br>**Social and emotional support**<br>**Fostering relationships**<br><br>**VOLUNTEER SUPPORT**<br>**Mentoring and ongoing volunteer support**
22	Palliative care volunteerism across the healthcare system: A survey study.	Palliative Medicine. 32(7):1233-1245, 2018 Jul.	Vanderstichelen S<br><br>Houttekier D<br><br>Cohen J<br><br>Van Wesemael Y<br><br>Deliens L<br><br>Chambaere K	Volunteers; home care services; medical oncology; nursing homes; palliative care; surveys and questionnaires	BACKGROUND: Volunteers fulfil several roles in supporting terminally ill people and their relatives and can positively influence quality of care. Healthcare in many countries faces resource constraints and some governments now expect communities to provide an increasing proportion of palliative care. However, systematic insights into volunteer presence, tasks and training and organisational challenges for volunteerism are lacking.<br><br>AIM: Describe organised volunteerism in palliative direct patient care across the Flemish healthcare system (Belgium).<br><br>DESIGN: A cross-sectional postal survey using a self-developed questionnaire was conducted with 342 healthcare organisations.	**ORGANISATIONAL/IMPLEMENTATION**<br>**Embedding the volunteer service within the organisation**<br>**Organisational barriers/expectations for volunteer roles**<br>**Support frameworks to train and support volunteers**<br><br>**VOLUNTEER ROLE**<br>**Engaging in advance care planning conversations**<br>**Tensions between professional vs volunteer roles**

					SETTING/PARTICIPANTS: The study included full population samples of palliative care units, palliative day-care centres, palliative home care teams, medical oncology departments, sitting services, community home care services and a random sample of nursing homes.<br><br>RESULTS: Responses were obtained for 254 (79%) organisations; 80% have volunteers providing direct patient care. Psychosocial, signalling and existential care tasks were the most prevalent volunteer tasks. The most cited organisational barriers were finding suitable (84%) and new (80%) volunteers; 33% of organisations offered obligatory training (75% dedicated palliative care, 12% nursing homes). Differences in volunteer use were associated with training needs and prevalence of organisational barriers.<br><br>CONCLUSION: Results suggest potential for larger volunteer contingents. The necessity of volunteer support and training and organisational coordination of recruitment efforts is emphasised. Organisations are encouraged to invest in adequate volunteer support and training. The potential of shared frameworks for recruitment and training of volunteers is discussed. Future research should study volunteerism at the volunteer level to contrast with organisational data.	
23	Developing a Palliative Care Competency Framework for Health Professionals and Volunteers: The	Journal of Palliative Medicine. 21(7):947-955, 2018 Jul.	McCallum M<br><br>Carver J<br><br>Dupere D	competency; education; framework; interprofessional; palliative care	BACKGROUND AND OBJECTIVES: In 2014, Nova Scotia released a provincial palliative care strategy and implementation working groups were established. The Capacity Building and Practice Change Working Group, comprised of health professionals, public	**COMPETENCIES FOR PALLIATIVE CARE VOLUNTEERS**<br>Although not specifically articulated which competencies/educational requirements pertain explicitly to volunteers – there is a

	Nova Scotian Experience.		Ganong S<br><br>Henderson JD<br><br>McKim A<br><br>McNeil-Campbell L<br><br>Richardson H<br><br>Simpson J<br><br>Tschupruk C<br><br>Jewers H		advisors, academics, educators, and a volunteer supervisor, was asked to select palliative care education programs for health professionals and volunteers. The first step in achieving this mandate was to establish competencies for health professionals and volunteers caring for patients with life-limiting illness and their families and those specializing in palliative care.<br><br>METHODS: In 2015, a literature search for palliative care competencies and an environmental scan of related education programs were conducted. The Irish Palliative Care Competence Framework serves as the foundation of the Nova Scotia Palliative Care Competency Framework. Additional disciplines and competencies were added and any competencies not specific to palliative care were removed. To highlight interprofessional practice, the framework illustrates shared and discipline-specific competencies. Stakeholders were asked to validate the framework and map the competencies to educational programs. Numerous rounds of review refined the framework.<br><br>RESULTS: The framework includes competencies for 22 disciplines, 9 nursing specialties, and 4 physician specialties.<br><br>CONCLUSIONS: The framework, released in 2017, and the selection and implementation of education programs were a significant undertaking. The framework will support the implementation of the Nova Scotia Integrated Palliative Care Strategy, enhance the interprofessional nature of palliative care, and	general list of 'competencies' set out in the framework:<br><br>**Principles of palliative care**<br><br>**Cultural safety**<br><br>**Communication**<br><br>**Optimising comfort and quality of life**<br><br>**Care planning and collaborative practice**<br><br>**Last days and hours**<br>*Patient and family care needs unique to the last days and hours of the patient's life.*<br><br>**Loss, grief, and bereavement**<br><br>**Professional and ethical practice**<br><br>**Self-care**<br><br>**Last days and hours**<br>*The unique care provided during the last days and hours of life*.<br><br>**Education**<br><br>**Evaluation**<br><br>**Research**<br><br>**Advocacy**

					guide the further implementation of education programs. Other jurisdictions have expressed considerable interest in the framework.	
24	Training and supportive programs for palliative care volunteers in community settings. [Review]	Cochrane Database of Systematic Reviews. (7):CD009500, 2015 Jul 20	Horey D<br><br>Street AF<br><br>O'Connor M<br><br>Peters L<br><br>Lee SF		BACKGROUND: Palliative care is specialised health care to support people living with a terminal illness and their families. The involvement of volunteers can extend the range of activities offered by palliative care services, particularly for those living in the community. Activities undertaken by palliative care volunteers vary considerably but can be practical, social or emotional in nature. The types of training and support provided to these volunteers are likely to affect the volunteers' effectiveness in their role and influence the quality of care provided to palliative care clients and their families. Training and support can also have considerable resource implications for palliative care organisations, which makes it important to know how to provide this training and support as effectively as possible.<br><br>OBJECTIVES: To assess the effects of training and support strategies for palliative care volunteers on palliative care clients and their families, volunteers and service quality.<br><br>SEARCH METHODS: We searched the Cochrane Central Register of Controlled Trials (CENTRAL, The Cochrane Library, 28 April 2014); MEDLINE (1946 to 28 April 2014); EMBASE (1988 to 28 April 2014); PsycINFO (1806 to 28 April 2014); CINAHL (EbscoHOST) (1981 to 28 April 2014); ProQuest Dissertations and Theses (1861 to	**ADVOCACY FOR THE DEVELOPMENT OF VOLUNTEER PROGRAMMES THAT ARE WELL EVALUATED.**<br><br>“Despite the wide use of volunteers in palliative care in the community and the associated investment to train and support them, there are no well designed studies that provide good evidence to guide palliative care volunteer training and support to ensure good outcomes for palliative care patients and their families, and for palliative care volunteers, or value for palliative care services.”

					28 April 2014). We also searched the Database of Abstracts of Reviews of Effects (DARE, The Cochrane Library); reference lists of relevant studies; and conducted an extensive search for evaluations published in government reports and other grey literature including the CareSearch database (www.caresearch.com.au (September 2004 to February 2012) and websites of relevant organisations, for unpublished and ongoing studies.<br><br>SELECTION CRITERIA: Randomised controlled trials (RCTs), quasi-randomised controlled trials, controlled before-and-after (CBA) studies and interrupted time series (ITS) studies of all formal training and support programs for palliative care volunteers. Programs or strategies in included studies were classified according to any stated or implied purpose: that is, whether they intended to build skills for the volunteer's role, to enhance their coping, or to maintain service standards.<br><br>DATA COLLECTION AND ANALYSIS: Two review authors screened 2614 citations identified through the electronic searches after duplicates were removed. The search of grey literature through websites yielded no additional titles. We identified 28 potentially relevant titles but found no studies eligible for inclusion.<br><br>MAIN RESULTS: We did not find any studies that assessed the effects of training and support strategies for palliative care volunteers that meet our inclusion criteria. The excluded studies suggest that trials in this	

					area are possible.<br><br>AUTHORS' CONCLUSIONS: The use of palliative care volunteers is likely to continue, but there is an absence of evidence to show how best to train or support them whilst maintaining standards of care for palliative care patients and their families.	
25	The Impact of Unusual End-of-Life Phenomena on Hospice Palliative Care Volunteers and Their Perceived Needs for Training to Respond to Them.	Omega - Journal of Death & Dying. :30222818788238, 2018 Jan 01	Claxton-Oldfield S<br><br>Gallant M<br><br>Claxton-Oldfield J	hospice; impact; palliative care; training; unusual end-of-life phenomena; volunteers	Thirty-nine hospice palliative care volunteers completed a survey examining (a) their beliefs about end-of-life phenomena (EOLP), (b) the impact of EOLP on their lives, and (c) their perceived needs for training to respond to them. Forty-nine percent of the volunteers either had personally witnessed an EOLP in their volunteer work and/or had a patient or patient's family member report an EOLP to them. More than half of the volunteers strongly agreed or agreed that EOLP have influenced their religious beliefs and their spirituality in a positive way (53% and 59%, respectively). Eighty-nine percent of the volunteers indicated that they had never received any training about EOLP, and nearly all of the volunteers were interested in learning more about EOLP. After completing the survey, 59% of the volunteers shared stories about EOLP they had either personally witnessed or been told about. The most frequently reported experiences involved deathbed visions. The implications of these findings are discussed.	**END OF LIFE PHENOMENA**<br>**Understanding of EOLP**<br>**Knowledge of EOLP**<br><br>“Based on the findings from this study, it is recommended that hospice palliative care volunteer training programs should include a module on the topic of EOLP. The module should:<br>• Help volunteers recognize and put a name to the different EOLP that often occur around the time of death and include concrete examples of each phenomenon.<br>• Include information about the prevalence (or frequency) of different EOLP.<br>• Provide information regarding the impact of EOLP on patients, the patients’ families, medical and nursing home staff, and hospice palliative care volunteers.<br>• Provide information on how to respond to patients’, patients’ family members’, and others’ reports of EOLP (i.e., knowing what to say and how to talk to patients or families about EOLP) in a way that is validating, normalizing, and supportive.”

26	Nationwide survey on volunteers' training in hospice and palliative care in Poland.	BMJ supportive & palliative care. 2016 Jul 29	Pawlowski L<br><br>Lichodziejewska-Niemierko M<br><br>Pawlowska I<br><br>Leppert W<br><br>Mroz P	Hospice care; Voluntary workers; education; palliative care; training programs	BACKGROUND: Volunteers working in hospice and palliative care facilities in Poland undertake various activities which are performed in accordance with legal regulations and the individual policies of each hospice. The aim of this study was to explore the roles and training of volunteers working in hospice and palliative care settings.<br><br>METHODS: A cross-sectional survey was carried out that investigated the services performed by volunteers and their preparation for work within residential hospices. Questionnaires were distributed to volunteers and hospice representatives, and the responses obtained underwent statistical	**VOLUNTEER TRAINING**<br>**Importance of volunteer training**<br>"All of the surveyed facilities responded that they required and expected some form of training from candidates in order to become volunteers"<br><br>**Specific training for direct patient contact**<br>**Basic understanding of palliative care**<br><br>**Theoretical and practical training**<br>"In 25 of the 28 examined units, volunteers participated in theoretical and practical training, which prepared them to support patients."<br><br>**Theoretical Training:**

					analysis.<br><br>PARTICIPANTS: A total of 180 volunteers and 28 hospice representatives from 29 residential hospices participated in this survey.<br><br>RESULTS: All hospices surveyed were supported by volunteers. 79% of volunteers worked alongside patients and performed the following services: accompanying patients (76%), feeding patients (61%), cleaning rooms (48%), dressing and bathing (42%) and organising leisure time (40%). Fewer volunteers were involved in activities outside of patient support-for example, charity work and fundraising (34%), cleaning hospice buildings (23%) as well as providing information and education (22%). According to volunteers, prior to undertaking their duties, 64% participated in theoretical training and 37% took part in a practical course. The majority attended courses relating to general knowledge of hospice and palliative care (64%) and volunteer rights and duties (55%).<br><br>CONCLUSIONS: Overall, proper training was an essential requirement needed to be fulfilled by volunteers, particularly when involved in direct patient support. Most volunteers were simultaneously involved in various areas of service; therefore, their training should be comprehensive.	**Psychological issues of hospice and Palliative care**<br>**General knowledge of palliative medicine**<br>**Volunteers' rights and duties**<br>**Spiritual care**<br>**Social support and pharmacotherapy**<br>**Rehabilitation in hospice and palliative care**<br>**Health and safety regulations**<br>**Hospice ideas and occupational therapy**<br><br>**Practical training:**<br>**Communication with patients**<br>**Nursing**<br>**Physiotherapy in palliative care**<br>**Assisting patients in moving from a bed to a wheelchair**<br><br>**VOLUNTEER SUPPORT**<br>**Ongoing training opportunities**
27	Personally Meaningful Rituals: A Way to Increase Compassion and Decrease Burnout among Hospice Staff and Volunteers.	Journal of Palliative Medicine. 19(10):1043-1050, 2016 Oct.	Montross-Thomas LP<br><br>Scheiber C<br><br>Meier EA		BACKGROUND: Rituals can increase a sense of connectedness, meaning, and support, especially after the death of those for whom we care. Hospice staff may benefit from the use of personal rituals as they cope with the frequent deaths of their patients, ultimately aiming to provide compassionate care while	“Of those hospice staff and volunteers who responded to the question, the majority reported using some form of personally meaningful ritual after the death of their patients (71%, n = 248).”

			Irwin SA		minimizing burnout.<br><br>OBJECTIVE: This study investigated the role of personally meaningful rituals in increasing compassion and decreasing burnout among hospice staff and volunteers.<br><br>DESIGN AND MEASUREMENTS: An online survey was completed by members of the National Hospice and Palliative Care Organization (NHPCO) which inquired about personal ritual practices, and included the Professional Quality of Life (ProQOL) scale to measure current levels of Compassion Satisfaction, Burnout, and Secondary Traumatic Stress.<br><br>SETTING/SUBJECTS: Three hundred ninety hospice staff and volunteers from across 38 states completed the online survey. The majority of participants were Caucasian and female, with an average of nine years of experience in hospice and palliative care.<br><br>RESULTS: The majority of hospice staff and volunteers used personally meaningful rituals after the death of their patients to help them cope (71%). Those who used rituals demonstrated significantly higher Compassion Satisfaction and significantly lower Burnout as measured by the ProQOL, with professional support, social support, and age playing significant roles as well.<br><br>CONCLUSIONS: Rituals may be an important way to increase compassion and decrease burnout among hospice staff and volunteers. Organizations may benefit from providing training and support for personalized rituals	“hospice staff and volunteers who engaged in rituals had significantly higher scores on Compassion Satisfaction... [and] significantly lower scores on burnout”<br><br>**VOLUNTEER SUPPORT**<br>**Rituals and ways to honour the lives of patients:** Understanding and techniques.

					among team members, especially new staff who may be at greater risk for burnout.	
28	'End of life could be on any ward really': A qualitative study of hospital volunteers' end-of-life care training needs and learning preferences.	Palliative Medicine. 31(9):842-852, 2017 Oct.	Brighton LJ<br><br>Koffman J<br><br>Robinson V<br><br>Khan SA<br><br>George R<br><br>Burman R<br><br>Selman LE	Volunteers; education; hospital volunteers; palliative care; qualitative research; terminal care	BACKGROUND: Over half of all deaths in Europe occur in hospital, a location associated with many complaints. Initiatives to improve inpatient end-of-life care are therefore a priority. In England, over 78,000 volunteers provide a potentially cost-effective resource to hospitals. Many work with people who are dying and their families, yet little is known about their training in end-of-life care.<br><br>AIMS: To explore hospital volunteers' end-of-life care training needs and learning preferences, and the acceptability of training evaluation methods.<br><br>DESIGN: Qualitative focus groups.<br><br>SETTING/PARTICIPANTS: Volunteers from a large teaching hospital were purposively sampled.<br><br>RESULTS: Five focus groups were conducted with 25 hospital volunteers (aged 19-80 years). Four themes emerged as follows: preparation for the volunteering role, training needs, training preferences and evaluation preferences. Many described encounters with patients with life-threatening illness and their families. Perceived training needs in end-of-life care included communication skills, grief and bereavement, spiritual diversity, common symptoms, and self-care. Volunteers valued	**VOLUNTEER ROLE**<br>**Advanced communication and listening skills**<br><br>**Aspects of end-of-life care (e.g. grief and bereavement, spiritual diversity, common symptoms, and self-care)**<br><br>**VOLUNTEER IDENTIFIED TRAINING NEEDS**<br>Volunteers in this study identified five distinct training needs related to working with patients and their families towards the end of life:<br>**1. communication skills**<br><br>**2. understanding grief and bereavement,**<br><br>**3. understanding spiritual diversity**<br><br>**4. understanding common symptoms at the end of life**<br><br>**5. volunteers' self-care**<br><br>**DELIVERY OF TRAINING**<br>**Simulated experience: Role plays,**<br>"Volunteers prefer training that is diverse and uses interactive teaching methods, drawing on real-life case examples and role plays"<br><br>**Online training:** volunteers warned against this as a method.

					learning from peers and end-of-life care specialists using interactive teaching methods including real-case examples and role plays. A chance to 'refresh' training at a later date was suggested to enhance learning. Evaluation through self-reports or observations were acceptable, but ratings by patients, families and staff were thought to be pragmatically unsuitable owing to sporadic contact with each.<br><br>CONCLUSION: Gaps in end-of-life care training for hospital volunteers indicate scope to maximise on this resource. This evidence will inform development of training and evaluations which could better enable volunteers to make positive, cost-effective contributions to end-of-life care in hospitals.	**VOLUNTEER SUPPORT**<br>**Ongoing Refresher training and other ongoing educational opportunities**<br>"They also want opportunities to refresh and consolidate learning."<br><br>"Volunteers are open to the effects of training being evaluated using their own self-reports or observed behaviours, but highlighted potential limitations of patient, family and/or staff feedback as a form of evaluation owing to their sporadic contact with each."
29	A Survey of Hospice Volunteer Coordinators: Training Methods and Objectives of Current Hospice Volunteer Training Programs.	American Journal of Hospice & Palliative Medicine. 34(5):412-416, 2017 Jun.	Brock CM<br><br>Herndon CM	hospice; objectives; training program; volunteer; volunteer coordinator	INTRODUCTION: Currently more than 5800 hospice organizations operate in the United States.¹ Hospice organizations are required by the Centers for Medicare and Medicaid Services (CMS) to use volunteers for services provided to patients.² Although CMS regulates the amount of hours hospice volunteers should provide, there are currently no national requirements for objectives of training.³ The purpose of this study was to gather information from a sample of hospices regarding volunteer coordinator background, current training for volunteers, importance of training objectives, and any comments regarding additional objectives.<br><br>METHODS: Representative state hospice organizations were contacted by e-mail requesting their participation and distribution of the survey throughout their member	**VOLUNTEER TRAINING PROGRAMMES:**<br>• Av. 19 hours duration<br>• Live lecture preferred method<br><br>**VOLUNTEER ROLE**<br>Increased time was needed within their training programs to cover:<br><br>**Effective communication skills**<br>**Emotional/Spiritual patient needs**<br>**Family/caregiver support strategies**<br><br>**SPECIFIC OBJECTIVES/TRAINING TOPICS ASSESSED AS IMPORTANT:**<br><br>**Organisational Context**<br>• History of hospice care<br>• Philosophy of hospice care<br>• Comprehensive understanding of services offered by hospice

					hospices. The survey asked demographical questions, along with ratings of training components based on perceived level of importance and time spent on each objective.<br><br>RESULTS: A total of 90 surveys were received, and the response rate was undeterminable. Results showed the majority of hospices were nonprofit, had less than 100 currently trained volunteers, and maintained an average daily patient census of less than 50. Questions regarding training programs indicated that most use live lecture methods of approximately 19 hours or less in duration. Overall, responding hospice organizations agreed that all objectives surveyed were important in training volunteers.<br><br>CONCLUSION: The small number of respondents to this survey makes generalization nationwide difficult, however it is a strong starting point for the development of further surveys on hospice volunteer training and achieving a standardized set of training objectives and delivery methods.	• Understanding similarities/differences between palliative and hospice care<br>• Understanding of CMS Hospice<br>• Conditions of Participation<br>• (Legislation) Health Insurance Portability and Accountability Act (HIPAA) training, testing, compliance<br>**Psychosocial Support**<br>• Understanding the emotional, physical, social, and spiritual needs of persons at end of life<br>**Communication Skills**<br>• Effective communication skills when assisting a hospice patient and family<br>• Family, caregiver, and loved one support<br>**Care after Death and Bereavement**<br>• Needs upon patient death; emergency procedures, universal precautions<br>• Basic info/knowledge about grief and loss<br>**Palliative and End of Life Care**<br>• Describing a plan of care<br>• Understanding/overview of chronic and life-limiting illnesses<br>• Understanding of common myths surrounding artificial hydration & nutrition<br>• Understanding of common medications used for pain and symptom support<br>• Understanding of DNR orders, living wills, and power of attorney<br><br>**“**advisable to incorporate additional content on interacting with patients with dementia or cognitive decline into training programs.”<br><br>**TRAINING EVALUATION AND ASSESSMENT OF VOLUNTEERS**<br>**Summative Assessment following training:**

						"Volunteer trainees were required to complete a summative evaluation or assessment in 80% of the hospice organizations surveyed."
3<br>0	Optimization of Methods Verifying Volunteers' Ability to Provide Hospice Care.	Journal of Cancer Education. 33(2):470-476, 2018 Apr.	Szeliga M<br><br>Mirecka J	Hospice; Method of verification; Volunteer	The subject of the presented work was an attempt at optimization of the methods used for verification of the candidates for medical voluntary workers in a hospice and decreasing the danger of a negative influence of an incompetent volunteer on a person in a terminal stage of a disease and his or her relatives. The study was carried out in St. Lazarus Hospice in Krakow, Poland, and included 154 adult participants in four consecutive editions of "A course for volunteers - a guardian of the sick" organized by the hospice. In order to improve the recruitment of these workers, the hitherto methods of selection (an interview with the coordinator of volunteering and no less than 50% of attendance in classes of a preparatory course for volunteers") were expanded by additional instruments-the tests whose usefulness was examined in practice. Knowledge of candidates was tested with the use of a written examination which consisted of four open questions and an MCQ test comprising 31 questions. Practical abilities were checked by the Objective Structured Clinical Examination (OSCE). A reference point for the results of these tests was a hidden standardized long-term observation carried out during the subsequent work of the volunteers in the stationary ward in the hospice using the Amsterdam Attitude and Communication Scale (AACS). Among the tests used, the greatest value (confirmed by a quantitative and qualitative analysis) in predicting how a	"The presented study is the first attempt in Poland to find a standardized tool that could be useful for verification of hospice volunteers"<br><br>**TRAINING EVALUATION AND ASSESSMENT OF VOLUNTEERS**<br>**Simulated experience: OSCE Station:**<br>**"**introducing an exam, resembling the single OSCE station could be a valuable instrument significantly increasing the value of the procedure of verification of volunteers for work in a hospice."

					given person would cope with practical tasks and in contact with the sick and their relatives had a practical test of the OSCE type.	
31	Continuing professional development for volunteers working in palliative care in a tertiary care cancer institute in India: a cross-sectional observational study of educational needs.	Indian Journal of Palliative Care. 21(2):158-63, 2015 May-Aug.	Deodhar JK<br><br>Muckaden MA	Continuing professional development; Educational needs; Palliative care; Volunteers	CONTEXT: Training programs for volunteers prior to their working in palliative care are well-established in India. However, few studies report on continuing professional development programs for this group.<br><br>AIMS: To conduct a preliminary assessment of educational needs of volunteers working in palliative care for developing a structured formal continuing professional development program for this group.<br><br>SETTINGS AND DESIGN: Cross-sectional observational study conducted in the Department of Palliative Medicine of a tertiary care cancer institute in India.<br><br>MATERIALS AND METHODS: Participant volunteers completed a questionnaire, noting previous training, years of experience, and a comprehensive list of topics for inclusion in this program, rated in order of importance according to them.<br><br>STATISTICAL ANALYSIS USED: Descriptive statistics for overall data and Chi-square tests for categorical variables for group comparisons were applied using Statistical Package for Social Sciences version 18.	"Understanding palliative care volunteers' educational needs is essential for developing a structured formal continuing professional development program and should include self-care as a significant component."<br><br>**VOLUNTEER SUPERVSION**<br>**Ongoing supervision needs**<br>**Ongoing education opportunity**<br><br>**TRAINING NEEDS OF VOLUNTEERS**<br>**Communication** – dealing with children; working with elderly; addressing denial and collusion<br>**physical aspects of palliative care and dying** – general information about cancer and treatments; pain; fatigue; nutrition; general nursing care<br>**Psychological aspects –** Depression; complicated grief; aggression; suicidal ideation<br>**Spiritual-existential aspects** – meaning of life; understanding of different faiths and belief systems<br>Self-care – personal death awareness; goal setting; impact of grief on self; need for supervision

					RESULTS: Fourteen out of 17 volunteers completed the questionnaire, seven having 5-10-years experience in working in palliative care. A need for continuing professional development program was felt by all participants. Communication skills, more for children and elderly specific issues were given highest priority. Spiritual-existential aspects and self-care were rated lower in importance than psychological, physical, and social aspects in palliative care. More experienced volunteers (>5 years of experience) felt the need for self-care as a topic in the program than those with less (<5-years experience) (P < 0.05).<br><br>CONCLUSIONS: Understanding palliative care volunteers' educational needs is essential for developing a structured formal continuing professional development program and should include self-care as a significant component.	
32	Palliative Care Volunteers Have High Workload but No Burnout: A Questionnaire Survey from Tanzania.	Journal of Palliative Medicine. 2019 Jan 07	Poyhia R<br><br>Jaatinen A<br><br>Niemi-Murola L<br><br>Mtega A<br><br>Mpumilwa G	home-based care; palliative care; relationships; volunteer; vulnerability; women	BACKGROUND: In Africa, the core of home-based care (HBC) in the villages is provided by volunteer helpers, individuals chosen to provide both support to patients and important information to health officers. Yet, voluntary work in palliative care and the burden of being a volunteer have not been studied in Africa.<br><br>OBJECTIVE: To study the content and burden of volunteer work in the palliative home care of Ilembula District Designated Hospital	"The desire to help the poor and sick neighbors and the experience of reducing suffering motivated the most to be a volunteer."<br><br>"This study provided evidence of the high workload and dedication of the volunteers in palliative care in Tanzania. Volunteers were highly motivated in their work and they did not report burnout. Yet, the results of this study propose that coping strategies for dealing with suffering and death, as well as psychological

			Mmbando P		(IDDH), a secondary care institution in Tanzania.<br><br>DESIGN: A descriptive prospective study using semistructured and closed questionnaires.<br><br>SETTING/SUBJECTS: The modified Palliative Care Evaluation Tool Kit (PCETK) and Professional Quality-of-Life Scale (ProQOL) were used to study the work content and workload of 47 volunteers in the palliative HBC of IDDH. ProQOL was translated to Kiswahili. Fifty-seven health care professionals and students validated the translation. Factorial analysis and Cronbach's alphas were calculated for reliability.<br><br>RESULTS: Responses to PCETK and ProQOL were received from 34 (72%) to 20 (42%) volunteers, respectively. The Kiswahili translation of ProQSL appeared to be highly reliable. On average, a volunteer worked 20 hours/month and had 22 patients. The main activities included helping with daily tasks, preparing meals, assisting with transport, and reporting the patient's clinical condition to the health care officers. The volunteers reported high satisfaction ratings (average 4.2, standard deviations 0.38) and had higher scores than the validation group in the compassion fatigue scale (2.42 vs. 1.55, $p < 0.01$) but no burnout.<br><br>CONCLUSIONS: The volunteers had high commitment and workload. Even so, coping strategies for dealing with suffering and death should be better addressed in training.	support, should be added to the training and care of volunteers."<br><br>**VOLUNTEER SUPPORT**<br>**Self-care information and strategies**.<br>**Volunteer Support**: coping strategies to overcome the 'stressors' of the volunteering role.<br>**Coping Strategies for dealing with suffering and death**<br>**Psychological Support**

33	Running a Volunteer Program for Palliative Care in a Chinese Hospital: Our Practice and Experience.	Chinese Medical Sciences Journal. 33(4):216-220, 2018 12 30.	Ning XH<br><br>Li J<br><br>Xiang YR		The establishment and development of volunteer team are very important in the whole process of palliative care. The concept and practice of palliative care have been developed in Peking Union Medical College Hospital (PUMCH) since the end of 2012. Great progress has been made in different aspects. Volunteers play an extremely important role in the development of palliative care in PUMCH. The whole work began with the establishment of volunteer teams. This article introduces the process of the establishment and development of palliative care volunteer team in PUMCH, aiming to provide practical references for hospitals in mainland China to develop their own palliative care volunteer team.	**TRAINING PROGRAMME**<br>• **4 hours**<br>• **2-4 hours shadowing 'senior' volunteers**<br><br>**VOLUNTEER ROLE**<br>**Concepts of Hospice Care**<br><br>**Role of Volunteers in a team**<br><br>**How to reach and help patients**<br><br>**Management**<br><br>**DELIVERY OF TRAINING**<br>**Simulated experience: Role Plays**
34	Perceptions of trained laypersons in end-of-life or advance care planning conversations: a qualitative meta-synthesis. [Review]	BMC Palliative Care. 17(1):98, 2018 Aug 06.	Somes E<br><br>Dukes J<br><br>Brungardt A<br><br>Jordan S<br><br>DeSanto K<br><br>Jones CD<br><br>Sanghvi UJ<br><br>Breathett K<br><br>Jones J<br><br>Lum HD	Advance care planning; Communication; Hospice care; Lay health navigators; Palliative care; Peer educators; Terminal care; Volunteers	BACKGROUND: Laypersons including volunteers, community health navigators, or peer educators provide important support to individuals with serious illnesses in community or healthcare settings. The experiences of laypersons in communication with seriously ill peers is unknown.<br><br>METHODS: We performed an ENTREQ-guided qualitative meta-synthesis. We conducted a systematic search of MEDLINE, PsycINFO, CINAHL, Cochrane Library, and AMED to include qualitative studies with data regarding communication and laypersons in advance care planning, palliative care, or end-of-life settings. Study quality was appraised using a standardized tool. The analysis identified key domains and associated themes relating specifically to laypersons' perspectives on communication.<br><br>RESULTS: Of 877 articles, nine studies	**VOLUNTEER ROLE**<br>**Advance Care Planning – supporting conversations around future care planning and end-of-life care issues**<br>"Laypersons specifically described increased awareness of end-of-life issues for themselves and, in turn, initiated conversations with families, friends, and sometimes their broader community."<br><br>"Since volunteers are more likely to commit to an activity that is personally satisfying, volunteer laypersons may constitute a reliable and cost-effective way to enhance advance care planning efforts and support individuals with palliative care needs."<br><br>**Communication Skills:**<br>• Listening Skills and responding to patient and family emotions<br>• Discussing death and death anxiety

					provided layperson quotations related to layperson-to-peer communication associated with advance care planning (n = 4) or end-of-life conversations (n = 5). The studies were conducted in United Kingdom (n = 4) or United States settings (n = 5). The synthesis of layperson perspectives yielded five main domains: 1) layperson-to-peer communication, focusing on the experience of talking with peers, 2) layperson-to-peer interpersonal interactions, focusing on the entire interaction between the layperson and peers, excluding communication-related issues, 3) personal impact on the layperson, 4) layperson contributions, and 5) layperson training. Laypersons described using specific communication skills including the ability to build rapport, discuss sensitive issues, listen and allow silence, and respond to emotions.<br><br>CONCLUSIONS: Published studies described experiences of trained laypersons in conversations with peers related to advance care planning or end-of-life situations. Based on these layperson perspectives related to communication, programs should next evaluate the potential impact of laypersons in meaningful conversations.	**VOLUNTEER SUPPORT**<br>**Self Care:** Techniques and coping<br><br>**Ongoing mentoring and support:** include meetings over time, supervision<br><br>**Formal structured supervision with Feedback**: identified as a learning need.<br><br>**DELIVERY OF TRAINING**<br>**Simulated Experience:** experiential learning, role plays and group-based learning<br><br>**Ongoing training and 'updates'**: training that carries on past the commencement of volunteering.

35	Hospice Palliative Care Volunteers as Program and Patient/Family Advocates.	American Journal of Hospice & Palliative Medicine. 34(9):844-848, 2017 Nov.	Claxton-Oldfield S<br><br>Blacklock K	advocacy; families; hospice; palliative care; patients; programs; volunteers	The objectives of this study were to examine (1) the extent to which hospice palliative care volunteers are involved in program and patient/family advocacy, (2) volunteers' willingness to engage in program and patient/family advocacy, and (3) volunteers' perceived needs for training on how to be an effective advocate. Thirty-four hospice palliative care volunteers responded to the survey developed for this study. The majority of the volunteers surveyed consider themselves advocates for their programs and many of those, who have not already done so, would be willing to promote their program (eg, give a community presentation, talk to local media) if asked. Half of the volunteers were aware of unmet needs of the patients/families they supported, and just over one-third wanted to advocate on behalf of their patients/families but did not know what to do or where to go. Recommendations for volunteer training are made.	**VOLUNTEER ROLE**<br>**Patient/Family Advocacy Issues:** "Because of their position outside the family and health-care professional roles, volunteers may become aware of patient/family needs that are not being met, and as a result, they have the potential to step in to advocate for those needs or to support the patient/family in advocating for themselves."<br><br>**Community Engagement and Advocacy for Volunteer Programme**:<br>"because of their experiences as volunteers (eg, seeing first hand the differences they can make in someone's life), volunteers are also in a position to be effective advocates for their volunteer program. This could include giving a presentation about their program in the community, participating in the training of new volunteers, talking to the local media, and being involved in fundraising events."
36	The liminal space palliative care volunteers occupy and their roles within it: a qualitative study.	BMJ supportive & palliative care. 2018 Dec 07	Vanderstichelen S<br><br>Cohen J<br><br>Van Wesemael Y<br><br>Deliens L<br><br>Chambaere K	end of life care; home care; palliative care; psychological care; social care; volunteering	OBJECTIVES: Volunteers have an important place in palliative care (PC), positively influencing quality of care for seriously ill people and those close to them and providing a link to the community. However, it is not well understood where volunteers fit into PC provision or how to support them adequately. We therefore chose to describe volunteer roles across care settings through the perspective of those closely involved in the care of terminally ill people.<br><br>METHODS: A qualitative study was conducted using both focus groups with volunteers, nurses, psychologists and family	**VOLUNTEER ROLE**<br>**Patient/Family Advocacy Issues:**<br>"Volunteers occupy a liminal space between the professional and the family domain, through which they notice and communicate patient needs missed by other caregivers."<br><br>**Communication Skills**<br><br>**Fostering and building relationships**<br>"Volunteers represent a more approachable face of care, focused on psychological, social and existential care and building relationships."<br><br>**VOLUNTEER SUPPORT**

					physicians and individual semistructured interviews with patients and family caregivers. Participants were recruited from hospital, home, day care and live-in services.<br><br>RESULTS: 79 people participated in the study. Two volunteer roles were identified. The first was 'being there' for the dying person. Volunteers represent a more approachable face of care, focused on psychological, social and existential care and building relationships. The second was the 'liaison' role. Volunteers occupy a liminal space between the professional and the family domain, through which they notice and communicate patient needs missed by other caregivers. Patient-volunteer matching was a facilitator for role performance; barriers were lack of communication opportunities with professional caregivers and lack of volunteer coordination.<br><br>CONCLUSION: Volunteers complement professional caregivers by (1) occupying a unique space between professionals, family and patients and fulfilling a liaison function and (2) being a unique face of care for patients. Healthcare services and policy can support volunteer role performance by ensuring frequent communication opportunities and volunteer coordination.	**Volunteer Support**: **regular ongoing mentoring.**<br>Healthcare services and policy can support volunteer role performance by ensuring frequent communication opportunities and volunteer coordination

37	Is There a Place for Humor in Hospice Palliative Care? Volunteers Say "Yes"!.	American Journal of Hospice & Palliative Medicine. 34(5):417-422, 2017 Jun.	Claxton-Oldfield S<br><br>Bhatt A	hospice; humor; laughter; palliative care; patients; volunteers	A survey was conducted to examine the frequency, acceptability, and functions of humor between hospice palliative care volunteers and their patients, from the volunteers' perspective. Thirty-two volunteers completed the survey, which was developed for this study. The results revealed that most patients and volunteers initiated humor either "often" or "sometimes" in their interactions. Over half of the volunteers considered humor to be either "very important" or "extremely important" in their interactions with patients (42% and 13%, respectively), with the patient being the determining factor as to whether and when it is appropriate or not (ie, volunteers take their lead from their patients). Volunteers mentioned a number of functions that humor serves within their patient interactions (eg, to relieve tension, to foster relationships/connections, and to distract). Laughter and humor fulfills one of the main goals of hospice palliative care, namely, improving patients' overall quality of life.	**VOLUNTEER ROLE**<br>**Communication Skills; use of humour in patient/volunteer interactions:**<br>“humour definitely has a place in hospice palliative care, with the patient being the determining factor as to whether and when it is appropriate or not (ie, volunteers must take their lead from, and be adept at, reading their patients).”
38	'Being with' or 'doing for'? How the role of an end-of-life volunteer befriender can impact patient wellbeing: interviews from a multiple qualitative case study (ELSA).	Supportive Care in Cancer. 26(9):3163-3172, 2018 Sep.	Dodd S<br><br>Hill M<br><br>Ockenden N<br><br>Algorta GP<br><br>Payne S<br><br>Preston N<br><br>Walshe C	End of life; Palliative care; Qualitative research; Volunteers	PURPOSE: To explore the perspectives of people anticipated to be in their last year of life, family carers, volunteers and staff on the impacts of receiving a volunteer-provided befriending service. Patient participants received up to 12 weeks of a volunteer-provided befriending intervention. Typically, this involved one visit per week from a trained volunteer. Such services complement usual care and are hoped to enhance quality of life.<br><br>METHODS: Multiple case study design (n = 8). Cases were end-of-life befriending services in home and community settings including UK-based hospices (n = 6), an acute hospital (n = 1) and a charity providing	**VOLUNTEER ROLE:**<br>**Communication Skills;**<br>• **building rapport, relationship building:**<br>• **Discussion of sensitive issues**<br>“An agreeable relationship between patient and volunteer and a degree of relational chemistry appeared an important prerequisite for impact. In practical terms, the relational aspects of the volunteer visit were apparent in the opportunity provided for conversation. Conversation was the most common object of patient’s appreciation.”

					support to those with substance abuse issues (n = 1). Data collection incorporated qualitative thematic interviews, observation and documentary analysis. Framework analysis facilitated within and across case pattern matching.<br><br>RESULTS: Eighty-four people participated across eight sites (cases), including patients (n = 23), carers (n = 3), volunteers (n = 24) and staff (n = 34). Interview data are reported here. Two main forms of input were described-'being there' and 'doing for'. 'Being there' encapsulated the importance of companionship and the relational dynamic between volunteer and patient. 'Doing for' described the process of meeting social needs such as being able to leave the house with the volunteer. These had impacts on wellbeing with people describing feeling less lonely, isolated, depressed and/or anxious.<br><br>CONCLUSION: Impacts from volunteer befriending or neighbour services may be achieved through volunteers taking a more practical/goal-based orientation to their role and/or taking a more relational and emotional orientation. Training of volunteers must equip them to be aware of these differing elements of the role and sensitive to when they may create most impact.	"Much attention has been given recently to concepts of community involvement in, and the importance of public health approaches to, palliative and end-of-life care [48]. Volunteer befriender services can be seen as part of a response to these calls."

39	To be a trained and supported volunteer in palliative care - a phenomenological study.	BMC Palliative Care. 16(1):18, 2017 Mar 14.	Soderhamn U<br><br>Flateland S<br><br>Fensli M<br><br>Skaar R	Benefits; Challenges; Clarified role; Community health care services; Followed-up; Meaningful experience; Phenomenology; Presence in volunteering; Qualitative research; Seriously ill	BACKGROUND: It has been found that including volunteers in palliative care is a positive contribution to seriously ill patients. It is, however, recommended that the volunteers are trained and supported. The aim of this study was to describe a group of trained and supported volunteers' lived experiences as volunteers in palliative care within the community health care services.<br><br>METHODS: This study adopted a descriptive phenomenological approach featuring individual interviews with nine volunteers. The interviews were analysed using the descriptive phenomenological research method according to Giorgi.<br><br>RESULTS: Being a volunteer in palliative care was both a positive and meaningful experience. It was a privilege being able to help those in need, which yielded positive returns. As a volunteer, it was important to be present for the ill persons and to follow them in their various physical and psychical states, which also implied that the volunteer had to face and deal with challenging situations. However, volunteers stated it was crucial to possess knowledge and life experience, as well as a clarified role, and they stressed the importance of being followed up by a mentor.<br><br>CONCLUSIONS: The findings showed that trained and supported volunteers among seriously ill or dying people within the realm of community health care services play an independent and important role in the palliative care team. A coordinator in palliative care is especially suitable for training and supporting the volunteers.	**Community Engagement and Advocacy for Volunteer Programme**:<br>"To ensure that palliative care volunteers are available in the future, it is important to inform individuals about the benefits of volunteering in palliative care within public forums. Likewise, health care personnel have to be aware of the volunteers' roles in palliative care."<br><br>**VOLUNTEER SUPPORT**<br>**Ongoing Support for volunteers**<br>**"**When volunteers experience their volunteering as meaningful, and when they receive recognition for performing voluntary tasks, they frequently wish to continue as volunteers.<br><br>**Community Engagement and Advocacy for Volunteer Programme**:<br>"To ensure that palliative care volunteers are available in the future, it is important to inform individuals about the benefits of volunteering in palliative care within public forums."<br><br>**ORGANISATIONAL/IMPLEMENTATION**<br>**Understanding of the volunteer role**<br>"health care personnel have to be aware of the volunteers' roles in palliative care."

40	Got volunteers? Association of hospice use of volunteers with bereaved family members' overall rating of the quality of end-of-life care.	Journal of Pain & Symptom Management. 39(3):502-6, 2010 Mar.	Block EM<br><br>Casarett DJ<br><br>Spence C<br><br>Gozalo P<br><br>Connor SR<br><br>Teno JM		CONTEXT: Volunteers are a key component of hospice, and they are required by Medicare conditions of participation in the United States. Yet, little is known about the impact of volunteers in hospice.<br><br>OBJECTIVES: The goal of this study was to characterize whether bereaved family members in hospice programs with increased use of volunteer hours per patient day report higher overall satisfaction with hospice services.<br><br>METHODS: A secondary analysis of the 2006 Family Evaluation of Hospice Care data repository with hospice organization data regarding the number of volunteer hours in direct patient care and the total number of patient days served. A multivariate model examined the association of institutional rate of bereaved family members stating end-of-life care was excellent with that of hospices' rate of volunteer hours per patient day, controlling for other organizational characteristics.<br><br>RESULTS: Three hundred five hospice programs (67% freestanding and 20.7% for profit) submitted 57,353 surveys in 2006 (54.2% female decedents and 47.4% with cancer). Hospice programs reported on average 0.71 hours per patient week (25th percentile: 0.245 hours per patient week; 75th percentile: 0.91 volunteer hours per patient week; and 99 th percentile: 3.3 hours per	**ORGANISATIONAL/IMPLEMENTATION**<br>**Recruitment and retention of volunteers**<br>A higher number of ‘Volunteer hours’ is related to higher relative satisfaction with care.<br><br>**VOLUNTEER SUPPORT**<br>**Ongoing support and mentoring for existing volunteers.**<br>Satisfaction with care was related to higher ‘volunteer hours’ – supporting current volunteers may promote longevity within the service.

					patient week). Those hospice programs in the highest quartile of volunteer usage had higher overall satisfaction compared with those in the lowest-quartile usage of volunteers (75.8% reported excellent overall quality of care compared with 67.8% reporting excellent in the lowest quartile. After adjustment for hospice program characteristics, hospice programs in the highest quartile had highest overall rating of the quality of care (coefficient=0.06, 95% confidence interval=0.04, 0.09).<br><br>CONCLUSION: In this cross-sectional study, hospice programs with higher use of volunteers per patient day were associated with bereaved family member reports that the hospice program quality of care was excellent.	
41	The use of reflective diaries in end of life training programmes: a study exploring the impact of self-reflection on the participants in a volunteer training programme.	BMC Palliative Care. 15:28, 2016 Mar 05.	Germain A<br><br>Nolan K<br><br>Doyle R<br><br>Mason S<br><br>Gambles M<br><br>Chen H<br><br>Smeding R		BACKGROUND: A training programme was developed and delivered to a cohort of volunteers who were preparing for a unique role to provide companionship to dying patients in the acute hospital setting. This comprehensive programme aimed to provide an opportunity for participants to fully understand the nature and responsibilities of the role, whilst also allowing sufficient time to assess the qualities and competencies of participants for their ongoing volunteering role. Participants completed reflective diaries throughout the training course to record their ongoing thoughts and feelings. The purpose	**‘BEING THERE’ AND ‘BEING PRESENT’ WITH THE PATIENT**<br>**spiritual and psychological components of what it means to provide a presence with another**<br><br>**VOLUNTEER SUPPORT**<br>**Self preservation and coping strategies**<br><br>**Personal loss and bereavement**<br><br>**Self Awareness**<br>Reflection and maintaining the emotional health and well being of the volunteering team.

			Ellershaw J		of this paper is to present a phenomenological analysis of these entries to understand participants' experiences, perceptions and motivations.<br><br>METHOD: The wider study was structured into three phases. Phase 1 was the delivery of a 12 week, bespoke training programme; Phase 2 involved a 26 week pilot implementation of the Care of the Dying Volunteer Service and Phase 3 was the research evaluation of the training and implementation which would inform the further development of the training programme. Self-reflection is a common component of End of Life training programmes and volunteers in this study completed a reflective diary after participation in each of the training sessions. A thematic analysis was undertaken to explore and understand the participants' experience, perceptions and motivations in relation to their participation in the training.<br><br>RESULTS: All 19 volunteers completed the reflective diaries. From a potential 228 diary entries over the 12 week training programme, 178 diary entries were submitted (78 %). The following key themes were identified: Dying Alone and the importance of being present, Personal loss and the reconstruction of meaning, Self-Awareness and Personal growth, Self-preservation and Coping strategies and group unity/cohesion.	

					CONCLUSIONS: The participants in this study demonstrated that they were able to use the diaries as an appropriate medium for reflection. Their reflections were also instrumental in the ongoing revision and development of the training programme. Analysis of their entries illustrated that the diaries could provide the opportunity for a reappraisal of their world view and personal philosophy around death and dying. Further research is undoubtedly required, however this paper suggests that self-reflection in this way, supports preparation in honing the appropriate attitudes and qualities required to work in this role.	
42	Spiritual care in the training of hospice volunteers in Germany.	Palliative & Supportive Care. 14(5):532-40, 2016 10.	Gratz M<br><br>Paal P<br><br>Emmelmann M<br><br>Roser T	Hospice volunteers; Palliative care; Spiritual care; Spirituality; Training program	OBJECTIVE: Hospice volunteers often encounter questions related to spirituality. It is unknown whether spiritual care receives a corresponding level of attention in their training. Our survey investigated the current practice of spiritual care training in Germany.<br><br>METHOD: An online survey sent to 1,332 hospice homecare services for adults in Germany was conducted during the summer of 2012. We employed the SPSS 21 software package for statistical evaluation.<br><br>RESULTS: All training programs included self-reflection on personal spirituality as obligatory. The definitions of spirituality used in programs differ considerably. The task of defining training objectives is randomly delegated to a	**SPIRITUALITY**<br>**Recognising spiritual diversity**<br>**Being aware of religious/spiritual needs of patients and their families**<br>**Supporting patients with diverse spiritual needs**<br>“This approach firmly rejects intentions to impose external standards of spirituality on recipients of hospice care. It also suggests a form of spiritual care that achieves expert management of the aspects of presence, listening, perception, acceptance, respect, and reaction.”<br><br>**PRESENCE**<br>**Being there and being with patients**<br>“spiritual care that achieves expert management of the aspects of presence, listening, perception, acceptance, respect, and reaction”

					supervisor, a trainer, or to the governing organization. More than half the institutions work in conjunction with an external trainer. These external trainers frequently have professional backgrounds in pastoral care/theology and/or in hospice/palliative care. While spiritual care receives great attention, the specific tasks it entails are rarely discussed. The response rate for our study was 25.0% (n = 332).<br><br>SIGNIFICANCE OF RESULTS: A need exists to develop training concepts that outline distinct contents, methods, and objectives. A prospective curriculum would have to provide assistance in the development of training programs. Moreover, it would need to be adaptable to the various concepts of spiritual care employed by the respective institutions and their hospice volunteers.	
43	Hospice Palliative Care Volunteers: A Review of Commonly Encountered Stressors, How They Cope With them, and Implications for Volunteer Training/Management. [Review]	American Journal of Hospice & Palliative Medicine. 33(2):201-4, 2016 Mar.	Claxton-Oldfield S	*coping; *hospice; *palliative care; *stressors; *volunteers	Hospice palliative care volunteer work--being with dying persons and their often distraught family members--has the potential to take an emotional toll on volunteers. The aim of this review article is to examine the types of stressors hospice palliative care volunteers typically experience in their work and how they cope with them. The results of this literature review suggest that hospice palliative care volunteers do not generally perceive their volunteer work as highly stressful. Nonetheless, a number of potential stressors and challenges were identified in the	**VOLUNTEER SUPPORT**<br>**Self-care information and strategies and personal resilience**<br><br>**Ongoing education and training/mentoring and support**<br><br>**Psychological Support and Coping**<br><br>**Strategies for dealing with suffering and death**<br><br>**ORGANISATIONAL/IMPLEMENTAITON**<br>**Avoiding role ambiguity**

					literature, along with some strategies that volunteers commonly employ to cope with them. The implications for volunteers and volunteer training/management are discussed. Copyright © The Author(s) 2015.	**ETHICAL ISSUES**<br>**Boundary issues**
44	Motivations, Satisfaction, and Fears of Death and Dying in Residential Hospice Volunteers: A Prospective Longitudinal Study.	American Journal of Hospice & Palliative Medicine. 33(4):335-9, 2016 May.	Nissim R<br><br>Emmerson D<br><br>O'Neill B<br><br>Marchington K<br><br>Draper H<br><br>Rodin G	fears of death and dying; hospice; motivations to volunteer; volunteers	RATIONALE: Studies conducted on hospice volunteers' characteristics and experiences have been cross-sectional. We conducted a prospective study to examine changes in the volunteer experience over time in a cohort of volunteers during the first year of a new residential hospice.<br><br>METHOD: Eighty-two active volunteers completed an online baseline survey, and of these, 39 completed a follow-up survey at 6 months. The survey included measures of motivations to volunteer, satisfaction with role and with the organization, and fear of death and dying.<br><br>RESULTS: Repeated measures analyses indicated that motivations to volunteer remained stable over time while volunteer satisfaction increased with time. Baseline level of fears of death and dying varied by age, volunteer role, and motivations to volunteer and decreased at 6 months.	**VOLUNTEER SUPPORT**<br>**Self-care information and strategies and personal resilience**<br><br>**Psychological Support Coping**<br><br>**Strategies for dealing with suffering and death**<br><br>**Death Anxiety**<br><br>**MOTIVATIONS FOR BEING A VOLUNTEER**<br>**Motivation assessment tools for selection criteria**

					CONCLUSION: Volunteering in a residential hospice tends to be a satisfying experience that helps to allay fears about death and dying.<br>Copyright © The Author(s) 2014.	
45	'A bridge to the hospice': the impact of a Community Volunteer Programme in Uganda.	Palliative Medicine. 25(7):706-15, 2011 Oct.	Jack BA<br><br>Kirton J<br><br>Birakurataki J<br><br>Merriman A		In Africa, the need for palliative care provision is escalating with an increasing number of people living with HIV/AIDS, coupled with rising cancer and AIDS-related cancer diagnoses. In Uganda there is a shortage of doctors, particularly in rural areas. To address this Hospice Africa Uganda developed a Community Volunteer Programme to train volunteers to help by providing support to patients in their own homes. The aim of this qualitative study was to evaluate the impact of the Community Volunteer Programme. Sixty-four interviews, with patients (21), community volunteer workers (CVWs) (32), and the hospice clinical teams (11) were conducted, using semi-structured digitally recorded individual, group and focus group interviews, at the Hospice Africa Uganda sites. The results reported the value of the Community Volunteer Programme, including the impact on patients and families, and how the CVWs acted as a 'bridge to the hospice' in identifying patients. Developing financial challenges that are emerging which could potentially impact	**VOLUNTEER TRAINING**<br>**Fundamentals of palliative care, HIV and cancer**<br>**Practical aspects of home nursing care (bed bathing, wound care, infection control, nutritional advice)**<br>**Communication skills**<br>**End-of-life care**<br>**Emotional support for carers**<br>**Bereavement support**<br>**Ethics of palliative care**<br><br>**VOLUNTEER SUPPORT**<br>**Ongoing opportunities for training**<br>**Ongoing mentoring/supervision**<br>**Reflective practice to aid support**<br>**Close link with clinical team**<br><br>**VOLUNTEER ROLE**<br>**Essential link between community and clinical care**<br>**Psychosocial support and counselling patients/family**

					on the programme were reported. The Community Volunteer Programme appears to be having a positive impact on patients, families and the hospice team, and is a model worthy of consideration by other developing countries to allow the expansion of palliative care.	
46	Ethical issues for hospice volunteers.	American Journal of Hospice & Palliative Medicine. 25(6):458-62, 2008 Dec-2009 Jan.	Berry P<br><br>Planalp S		Health care professionals usually receive professional education in ethics, but the half million hospice volunteers in the United States may receive only brief training that is limited to confidentiality and the volunteer role. The purpose of this study was to explore ethical issues hospice volunteers confront in their work. Interviews with 39 hospice volunteers were conducted, audio recorded, transcribed, and analyzed using qualitative methods. Prominent themes were dilemmas about gifts, patient care and family concerns, issues related to volunteer roles and boundaries, and issues surrounding suicide and hastening death. Suggestions for training include discussions of ethics after initial training once volunteers had confronted ethical issues, with special emphasis on strategies for negotiating their uneasy role positioned between health care professional and friend.	**ETHICAL ISSUES**<br>**Boundary issues.**<br>**Ethical issues and the volunteer role.**<br><br>**VOLUNTEER TRAINING AND SUPPORT**<br>**Ongoing support and mentoring.**

4 7	Imagine you are dying: would you be interested in having a hospice palliative care volunteer?.	American Journal of Hospice & Palliative Medicine. 26(1):47-51, 2009 Feb-Mar.	Claxton-Oldfield S<br><br>Gosselin N<br><br>Claxton-Oldfield J		A community sample of 100 adults was asked to imagine that they had been recently diagnosed with a life-threatening illness. After reading about the services provided by hospice palliative care volunteers, participants were asked whether they would choose to have a volunteer help them and to give a reason for their answer. Eighty-nine of the 100 participants indicated that they would use the help of a volunteer. The most commonly given reasons for utilizing a volunteer included for the general support they provide, help with practical things, and lack of family nearby; reasons given for declining the services of a volunteer included "I'm a private person" and "I don't need any help." Sixty-five participants knew that such a volunteer program was available. Of the 35 participants who did not, 31 (89%) expected their family doctor to tell them about it. The implications of these findings for volunteer program coordinators are discussed.	**VOLUNTEER ROLE**<br>**Practical aspects of support.**<br>**Defining the role of the volunteer within the organisation.**<br><br>**Emotional and social support**<br>**Being there for and being with patients**<br>**Providing practical support and respite.**<br>**Informational support or resources**<br>**Supporting the patient when family is not available.**<br>**Supporting family members**<br><br>**UNDERSTANDING THE INDIVIDUALITY**<br><br>**ORGANISATIONAL/IMPLEMENTATION**<br>**Organisational policies regarding hands on care provided by volunteers.**<br>**Knowledge and understanding of the role of the volunteer service across the organisation**
4 8	When to say "yes" and when to say "no": boundary issues for hospice palliative care volunteers.	American Journal of Hospice & Palliative Medicine. 28(6):429-34, 2011 Sep.	Claxton-Oldfield S<br><br>Gibbon L<br><br>Schmidt-Chamberlain K		A total of 79 hospice palliative care volunteers from 2 community-based hospice programs responded to a 27-item Boundary Issues Questionnaire that was specifically developed for this study. Volunteers were asked to indicate whether or not they considered each item (eg, "Lend personal belongings to a patient or family," "Agree to be a patient's power of attorney," "Attend/go into a patient's medical appointment") to be something they should not do and to indicate whether or not	**ROLE OF THE VOLUNTEER**<br>**Definitions of roles and responsibilities.**<br><br>**ETHICAL ISSUES IN THE VOLUNTEER ROLE**<br>**Boundary issues.**<br>**Preserving professional boundaries whilst fostering social relationships.**<br>**Accepting gifts and becoming 'involved'**<br>**Confidentiality within the volunteer role.**<br><br>**COMMUNICATION**

					they have ever done it. On the basis of the volunteers' responses, the authors distinguished between "definite boundary issues" (things volunteers should never do, for example, "Accept money from a patient or family"), "potential boundary issues" (things volunteers should stop and think twice about doing, for example, "Accept a gift from a patient or family"), and "questionable boundary issues" (things volunteers should be aware of doing, for example, "Give your home phone number to a patient or family"). The implications of these findings for training volunteers are discussed and the need for clear and unambiguous organizational policies and procedures to preserve boundaries is stressed. Without clear policies, etc, community-based hospice programs may be putting themselves at legal risk.	**Challenges of information sharing.**<br>**Collusion of patient/family.**<br><br>**ORGANISATIONAL/IMPLEMENTATION**<br>**Policies and procedures to establish role boundaries.**
49	Volunteer involvement in the organisation of palliative care: A survey study of the healthcare system in Flanders and Dutch-speaking Brussels, Belgium.	Health & Social Care in the Community. 27(2):459-471, 2019 Mar.	Vanderstichelen S<br><br>Cohen J<br><br>Van Wesemael Y<br><br>Deliens L<br><br>Chambaere K	Belgium; involvement; palliative care; surveys and questionnaires; volunteers	Ageing populations increasingly face chronic and terminal illnesses, emphasising the importance of palliative care and quality of life for terminally ill people. Facing resource constraints in professional healthcare, some governments expect informal caregivers like volunteers to assume a greater share of care provision. We know volunteers are present in palliative care and perform many roles, ranging from administration to providing companionship. However, we do not know how involved they are in the organisation of care and how healthcare organisations appraise their involvement. To address this,	**ORGANISATIONAL/IMPLEMENTATION**<br>**Investment in training volunteers**<br>**Valuing the contribution of volunteers as an organisation**<br>**Embedding the volunteer service within the organisational structures**<br><br>“Results suggest that generalist palliative care settings, in particular nursing homes, use a volunteer model with low involvement levels. Dedicated palliative care services and sitting services indicate the highest degrees of actual volunteer involvement. A previous study also indicated that nursing homes provide little training to their volunteers and had them perform fewer

					we provide an extensive description of the involvement of volunteers who provide direct patient palliative care across the Flemish healthcare system in Belgium. We conducted a cross-sectional postal survey of 342 healthcare organisations in Flanders and Brussels in 2016, including full-population samples of palliative care units, palliative day care centres, palliative home-care teams, medical oncology departments, sitting services, community home-care services, and a random sample of nursing homes. Volunteer involvement was measured using Sallnow and Paul's power-sharing model, which describes five hierarchical levels of engagement, ranging from being informed about the organisation of care to autonomy over certain aspects of care provision. Response was obtained for 254 (79%) organisations. Volunteers were often informed about and consulted regarding the organisation of care, but healthcare organisations did not wish for more autonomous forms of volunteer involvement. Three clusters of volunteer involvement were found: "strong involvement" (31.5%), "restricted involvement" (44%), and "uninvolved" (24.5%). Degree of involvement was found to be positively associated with volunteer training ($p < 0.001$) and performance of practical ($p < 0.001$) and psychosocial care tasks ($p < 0.001$). Dedicated palliative care services displayed a strong degree of volunteer involvement, contrary to generalist palliative care services,	tasks relative to other healthcare organisations (Vanderstichelen et al., 2018). These differences in involvement may therefore also be due to the lower emphasis on palliative care in these generalist care organisations, further emphasising the importance of volunteers to palliative care provision.”

					suggesting volunteers have a more important position in dedicated palliative care services. A link is found between volunteer involvement, training, and task performance. Copyright © 2018 John Wiley & Sons Ltd.	
50	Motivations of German Hospice Volunteers: How Do They Compare to Nonhospice Volunteers and US Hospice Volunteers?.	American Journal of Hospice & Palliative Medicine. 33(2):154-63, 2016 Mar.	Stelzer EM<br><br>Lang FR	hospice; motivation; palliative care; recruitment; retention; volunteering	We examined reasons of volunteering for hospice and nonhospice organizations in a study with 125 volunteers (22-93 years) from the United States and Germany. Motives of US and German hospice volunteers revealed similarities and few differences. Hospice volunteers are involved because they seek to help others, seek new learning experiences, seek social contacts, or seek personal growth. The US hospice volunteers reported motives related to altruistic concerns, enhancement, and social influence as more influential, while German hospice volunteers rated career expectations as being more important. Comparison of German hospice with nonhospice volunteers revealed stronger differences: German hospice volunteers scored higher on altruistic motives, while German nonhospice volunteers yielded higher scores on self-serving motives. Findings contribute to improved understanding of volunteering motivation and of activating or retaining hospice volunteers. Copyright © The Author(s) 2014.	**VOLUNTEER TRAINING**<br>**Ongoing opportunities for further education**<br>**Coping skills and self-care**<br>**Ongoing support and mentoring**<br><br>“lower scores on neuroticism might be highly beneficial for hospice volunteers because it allows them to stay calm in stressful situations, to deal with advanced illness or death, and to support the<br>bereaved, whereas high scores of neuroticism were associated with limited coping skills and fear of death”<br><br>**ORGANISAITONAL/IMPLEMENTATION**<br>**Motivation assessment tools for selection criteria**

					RETENTION OF VOLUNTEERS - CONTINUING EDUCATION	
51	Trends in the Use of Volunteers in US Hospices: 2000 to 2010.	American Journal of Hospice & Palliative Medicine. 33(3):256-63, 2016 Apr.	Apenteng BA<br><br>Linder DF<br><br>Opoku ST<br><br>Lawrence RH<br><br>Upchurch LA	Medicare Hospice Benefit; freestanding; hospice; trends; volunteers	PURPOSE: Using a longitudinal sample of freestanding Medicare-certified hospices in the 50 US states and the District of Columbia, this study sought to explore the factors associated with volunteer demand and describe how volunteer use has changed from 2000 to 2010.<br><br>RESULTS: A temporal decline in the extent of use of volunteers in freestanding hospices was observed over the study period. Findings indicated that both organizational and environmental factors influence the use of volunteers in US freestanding hospices.<br><br>CONCLUSION: Given the importance of volunteers, both in the preservation of hospices' philanthropic traditions and in reducing health care expenditure at the end of life, research is needed to further evaluate the factors associated with this decline. Emphasis should be placed on improving the retention of the existing hospice volunteer workforce.<br>RETENTION OF VOLUNTEERS -	**RETENTION OF VOLUNTEERS**<br>"Emphasis should be placed on improving the retention of the already existing hospice volunteer workforce. Hospices should adopt practices that improve volunteer morale and foster a sense of belongingness in the organization". Such as:<br><br>**VOLUNTEER SUPPORT**<br>**Ongoing support and mentoring of volunteers**<br>**Regular education or information sessions for volunteers,**<br>**Rituals and ways to honour the lives of patients:** Understanding and techniques.<br><br>**PROFESSIONAL/CLINICAL ROLES VS VOLUNTEER ROLES**<br>**Enhancing communication between staff and volunteers**<br>**Giving volunteers their own office space**

					CONTINUING EDUCATION<br>Copyright © The Author(s) 2014.	
5<br>2	Hospice palliative care volunteers: the benefits for patients, family caregivers, and the volunteers.	Palliative & Supportive Care. 13(3):809-13, 2015 Jun.	Claxton-Oldfield S	Benefits; Hospice; Palliative care; Volunteers	OBJECTIVE: Terminally ill patients and family caregivers can benefit greatly from the support and care provided by trained hospice palliative care volunteers. The benefits of doing this kind of volunteer work also extend to the volunteers themselves, who often say they receive more than they give from the patients/families they are "privileged" to be with. The purpose of this article is to demonstrate how hospice palliative care volunteerism benefits both the patients and families who utilize this service as well as the volunteers.<br><br>METHOD: A review of studies demonstrating how terminally ill patients, and especially family caregivers, can benefit from the use of hospice palliative care volunteers and how the volunteers themselves benefit from their experiences.<br><br>RESULTS: Terminally ill patients and families receive many benefits from using the services of hospice palliative care volunteers, including	**VOLUNTEER ROLE – Task vs Presence**<br>emotional support, companionship, and practical assistance (e.g., respite or breaks from caregiving)<br>**Being there**<br>**Doing for**

					emotional support, companionship, and practical assistance (e.g., respite or breaks from caregiving). Volunteering in hospice palliative care also provides many benefits for the volunteers, including being able to make a difference in the lives of others, personal growth, and greater appreciation of what is really important in life.<br><br>SIGNIFICANCE OF RESULTS: More needs to be done to promote the value of hospice palliative care volunteers to those who can really benefit from their support and care (i.e., patients and their families) as well as to help people recognize the potential rewards of being a hospice palliative care volunteer. It is a win-win situation.	
53	Motivations, Death Anxiety, and Empathy in Hospice Volunteers in France.	American Journal of Hospice & Palliative Medicine. 32(5):521-7, 2015 Aug.	Garbay M<br><br>Gay MC<br><br>Claxton-Oldfield S	death anxiety; empathy; hospice; hospice volunteer; palliative care	This study examined the motivations for volunteering of hospice volunteers in France. In addition, their levels of death anxiety and empathy were measured and compared with those of French non-hospice volunteers and non-volunteers. Three questionnaires-the Inventory of Motivations for Hospice Palliative Care Volunteerism (IMHPCV), the Templer/McMordie Death Anxiety Scale, and the Interpersonal Reactivity Index-were sent via an Internet link to 2 hospice volunteer associations and to non-hospice volunteers and non-volunteers (only the hospice volunteers received the IMHPCV). Altruistic motives had the most influence on the respondents' decision to become a hospice	**ONGOING SUPERVISION OF VOLUNTEERS**<br>**Coping strategies (volunteer role)**<br>**Coping with death and dying**<br>"Perhaps a measure of death competency, such a Bugen's Coping with Death Scale—a measure designed to assess people's ability to cope with death—might have revealed differences among the 3 groups in this study. Claxton-Oldfield, et al, for example, found that, after undergoing volunteer training, hospice volunteers reported feeling more able to cope with death and dying."<br><br>**Death Anxiety**<br>"The numbers of hospice volunteers, non-hospice volunteers, and non-volunteers who had experienced the death of a loved one were high".

					volunteer. French hospice volunteers scored significantly lower on 3 categories of motives on the IMHPCV compared to a sample of Canadian hospice palliative care volunteers (study 2), suggesting that cultural differences may be involved. No significant differences were found in levels of death anxiety or empathy between the 3 groups of respondents of the study. IMPLICATIONS FOR SCREENING OF POTENTIAL VOLS - DEATH ANXIETY AND COPING<br>Copyright © The Author(s) 2014.	**ORGANISAITONAL/IMPLEMENTATION**<br>**Motivation assessment tools for selection criteria**
54	Volunteers trained in palliative care at the hospital: an original and dynamic resource.	Palliative & Supportive Care. 13(3):601-7, 2015 Jun.	Delaloye S<br><br>Escher M<br><br>Luthy C<br><br>Piguet V<br><br>Dayer P<br><br>Cedraschi C	General hospitals; Palliative and supportive care; Palliative care volunteers; Role identification; Volunteer satisfaction	"OBJECTIVE: Volunteers trained in palliative care are increasingly present in acute care units in general hospitals. Nevertheless, there still are few available data on this topic, especially concerning the integration of volunteers outside the palliative structures. Our present study aimed to describe the experience of volunteers trained in palliative care in the context of a primary care hospital. In particular, the difficulties and the benefits of this specific position were evaluated according to volunteers' own perceptions and words.<br><br>METHOD: We employed a qualitative method. Various aspects of the volunteer's role were explored by means of semistructured questions, addressing their activity, their motivations, and their feelings. Participants were volunteers (n = 19) trained in palliative care and working at a university hospital. After giving written consent, they completed the	**VOLUNTEER ROLE**<br>**Role Definitions/boundaries**<br>“the position of volunteer was characterised as complex. This complexity was expressed in three main ways:<br>• the difficulty to integrate and explain one's role within the healthcare team and with patients<br>• the difficulties related to the necessity of constant adaptation to the shifting context of acute care wards (short hospitals stays, shifts in the team, lack of intimacy in the rooms)<br>• the difficulty to define precisely and definitively the limits of the position of volunteer”<br><br>**Developing and maintaining relationships**<br>“volunteers emphasized the ability to listen to and empathise with patients…the quality of support was essential in their relationships with patients”.<br>**MONITORING AND ONGOING SUPERVISION**

					semistructured questionnaire at home. Content analysis was used to identify the main categories of answers and the principal themes reported by the volunteers.<br><br>RESULTS: The main difficulties were related to uncertainty of the context. As every situation is different, volunteers could not define their role once and for all. However, they derived great satisfaction from their activity. A supporting frame and a good balance between constraints and autonomy were facilitating factors. Besides, the complexity related to the context contributed to make the position valuable and challenging.<br><br>SIGNIFICANCE OF RESULTS: Integrating a voluntary service in a primary care hospital is partly based on active participation of the volunteers in developing their position in a more adequate way. In return, this relative autonomy implies a rigorous and supportive attitude from the institution."	**Regular contact with other volunteers**<br>**Support from volunteer coordinators**<br>**Opportunity for reflection and reflexivity**<br>"…belonging to a well-defined group, with its rules, but also with the exchanges it allows, constituted a major criterion of satisfaction. The quality of the welcome, the support, and the user-friendliness of the group were often pointed out by volunteers as essential features."<br><br>**ORGANISATIONAL/IMPLEMENTATION**<br>**Embedding the volunteer service within the structures of the organisation**<br>"The complexity and ever-changing characteristics of the context clearly made for elements hard to overcome. It was also the major source of frustrations. However, these difficulties were also perceived as a challenge, which when taken up contributed to the value of volunteering:<br><br>It takes time to become integrated. (. . .) What I like most is the complicity and discussions I can have with the healthcare team. We help each other by exchanging our feelings and what we could do for the patient's well-being. It is often very constructive. (volunteer 18)"<br><br>**VOLUNTEERING IN HOSPITAL SETTING**<br>indicate that volunteers trained in palliative care have their place in acute and general care settings. The study constitutes a first contribution to better understand the potential benefits and specificities of volunteer work in this particular context.
