# Supplementary material for: ILIVE Project Volunteer study. Developing international consensus for a European Core Curriculum for hospital end-of-life-care volunteer services, to train volunteers to support patients in the last weeks of life: A Delphi study
Source: Palliat Med. 2021 Oct 20;36(4):652–70. doi: 10.1177/02692163211045305 (PMC9006393; doi:10.1177/02692163211045305)
Supplement: sj-pdf-2-pmj-10.1177_02692163211045305 – Supplemental material for ILIVE Project Volunteer study. Developing international consensus for a European Core Curriculum for hospital end-of-life-care volunteer services, to train volunteers to support patients in the last weeks of life: A Delphi study [file sj-pdf-2-pmj-10.1177_02692163211045305.pdf]

iLIVE Project: Delphi Study Round 1 Questionnaire

1. "BEING THERE" AND "BEING PRESENT" WITH THE PATIENT/FAMILY

This category relates to the concept of 'active relational skills', i.e. what characterises a 'good relationship' between patients and volunteers. For example, to 'be there' for someone takes unconditional acceptance, empathy, authenticity, warmth, understanding, sensitivity, honesty, involvement, respect, expertise, attention, enthusiasm. Training should enhance and hone these skills and qualities.

a. Establishing an environment of 'mutuality' to promote empathy and a 'non-judgemental' relationship[1-4]

b. Being attentive to the emotional needs of persons at the end of life (e.g. listening to the patient's/family's fears, worries, hopes, dreams, and other feelings etc) [2, 5-9]

c. Being responsive to the 'uniqueness of the other' [2, 5-9]

d. How to be 'present' with patients and families[3, 4, 10-12]

e. Relational attunement; establishing a connection, building rapport and relationship building with patients and families[1, 3, 13-18]

f. 'Journeying with' patients, sitting with patients in the last hours of life[4, 8, 11]

g. Providing social support to patients and their families (e.g. talking with patients/families, sharing hobbies and interests, reading to the patient etc) [5, 6, 10, 14, 16]

h. Understanding the social nature of the volunteer role [5, 6, 17]

i. Use of humour in patient/volunteer interactions[15]

2. COMMUNICATION SKILLS

This category relates to the 'Instrumental' elements which can underpin good communication skills, for example 'learned' communication skills that adhere to a 'formal' learning and teaching agenda. These are 'taught skills' which provide volunteer with a 'framework' to guide their communication and engagement with patients and families.

a. Advanced communication skills training: listening skills and responding to patient and family emotions[5, 13, 19-21]

b. Advanced communication skills training: barriers to effective communication[5, 19, 20]

c. Understanding of "Do Not Attempt Resuscitation" orders, living wills, and power of attorney[5]

d. Communication with patients with dementia and cognitive decline[5]

e. Communication skills for talking with children[22]

f. Dealing with issues of denial [13, 22]

g. Dealing with issues of collusion[13, 22]

h. General Introduction to Communication Skills: the need for good communication skills[20, 23, 24]

i. Communication skills to support conversations around future care planning (Advance Care Planning)[9, 21]

j. Communication skills to support conversations around end-of-life care issues [9, 21]

3. CULTURAL COMPETENCY

Cultural competence can be defined as the ability to understand, communicate with and effectively interact with people with diverse cultures, values, beliefs, feelings. Cultural competence encompasses being aware of one's own world view, developing positive attitudes towards cultural differences, gaining knowledge of different cultural practices and world views.

a. Understanding diversity and seeing patients and their families as individuals[9, 12, 25-28]

b. Understanding personal values, belief systems, attitudes, judgments and worldviews, and how these may impact on the care and support provided to patients and their families[9, 11, 25, 26]

c. How to support patients with diverse cultures, values, beliefs, and feelings[9, 25, 26]

d. Peer Support: Activities/resources to build and facilitate strong relationships with other volunteer colleagues, to discuss difficult situations or patients, to ask questions and give or receive advice in a friendly nonjudgmental environment[26, 29]

e. Exploration of fear of death and death anxiety[11, 23, 26, 30]

4. END OF LIFE PHENOMENA (EOLP)

End-of-life phenomena (EOLP) has been defined by Claxton-Oldfield as “unusual happenings that occur shortly before, at the time of, or shortly after a person dies”[31].

a. Knowledge and understanding of different EOLP[31]

b. Understanding the prevalence and impact of EOLP on patients [31]

c. How to offer support to patients and families regarding EOLP[31]

5. DEFINING AND PROMOTING UNDERSTANDING OF THE VOLUNTEER ROLE

This category relates to defining the role of the volunteer, in the care of patients in the last hours of life and their families. This refers to establishing definitions of role, practice and the volunteer 'place' within the organisation.

a. Understanding the 'definition' of the volunteer role within the service[3, 18, 29, 32]

b. Understanding the volunteer role as part of the care team[29, 33]

c. Understanding of the complexities of the care environment and the role of the volunteer within it; exploring power relationships between volunteer/staff and volunteer/patient and family[3, 9]

6. ETHICAL ISSUES RELATING TO END OF LIFE CARE AND THE VOLUNTEER ROLE

This category has been included to highlight the complexity of the volunteer role and the relationships that are built with patients, families and explore the potential ethical conflicts this could generate.

a. Issues of confidentiality and how to navigate this within the volunteer role[23, 34]

b. How to ensure confidentiality is upheld whilst undertaking the volunteer role[23]

c. Negotiating 'boundary spaces' within the role of a volunteer (e.g. not 'friend' or 'professional' and not 'paid' member of the organisation)[9, 10, 18, 34, 35]

d. Understanding of ethical Issues that could be encountered as part of the volunteer role (e.g. ethical dilemmas, competing interests, receiving gifts, clinical concerns etc) [11, 34, 35]

e. Understanding ethical issues in palliative and end of life care (e.g. assisted suicide, hastening death etc)[10, 35]

7. LOSS, GRIEF AND BEREAVEMENT

This category reflects the emotional impact of life-threatening illness and end of life on patients and families. Understanding loss and the diverse ways that people respond to loss may be pertinent for volunteers caring for patients at the end of life in the hospital setting.

a. Understanding grief[10, 19, 23, 28]

b. Understanding loss[10, 19, 23, 28]

c. Understanding bereavement[10, 19, 28]

d. Learning how to provide support to families, through grief and bereavement[6, 19, 36]

e. Understanding the nature and impact of 'Complicated Grief'[22]

f. Exploring the personal impact of grief and impact on the volunteer role[19, 22]

8. PHYSICAL SIGNS AND SYMPTOMS IN PALLIATIVE AND END OF LIFE CARE

This category reflects findings in the literature that suggest a basic knowledge of the common symptoms associated with life-limiting conditions, and signs and symptoms of approaching death, as potentially useful in reducing anxiety, whether their own, or that of patients or families.

a. Recognising changes in a patient's clinical condition[37]

b. Issues relating to patients in isolation due to disease/condition[37]

c. Understanding common symptoms at the end of life[5, 19, 23]

d. Understanding the physical needs of persons at the end of life (e.g. mobility, cognition, dysphasia etc)[5]

e. Understanding of issues of hydration at the end of life[5]

f. Understanding of issues of nutrition at the end of life[5]

g. Understanding of issues of artificial hydration at the end of life[5]

h. Understanding of issues of artificial nutrition at the end of life [5]

i. Understanding of common medications used for pain and symptom control[5]

j. Caring for 'actively dying' patients (e.g. days/hours leading up to death)[10]

k. Understanding of the physiology, signs and symptoms, of dying[10, 24, 36]

9. PRACTICAL ASPECTS OF THE VOLUNTEER ROLE (DELIVERING CARE AND SUPPORT)

Literature from this review highlighted a range of different 'practical' aspects of the volunteer role. While some volunteer services advocated for 'hands on' and 'direct' care from volunteers such as massage/touch, other services preferred volunteers to be involved in less direct care such as 'running errands'.

a. Comfort measures and strategies to support the patient (e.g. relaxation techniques, meditation, music/art therapy)[23, 37]

b. 'Hands on' comfort measures to provide comfort to the patients (e.g. touch, massage)[10, 36]

c. Establishing a process of 'handover' between volunteers to support continuity of care[37]

d. Providing practical support to patients and their families (e.g. running errands and responding to needs) [6, 12, 36]

e. Identification of patients/family in need of volunteer support[33, 38]

f. Practical care that can be delivered by the bedside (e.g. helping with eating, drinking, support with washing and cleaning teeth etc)[10]

10. PSYCHOLOGICAL/PSYCHOSOCIAL ASPECTS OF CARE AT THE END OF LIFE

This category reflects that for some volunteer services, psychosocial and existential elements of care have been highlighted as a core part of 'tasks' undertaken by volunteers. Ensuring volunteers are equipped to engage in this aspect of care necessitates increased volunteer training provision.

a. Issues regarding depression at the end of life[22]

b. Issues regarding anxiety at the end of life[22]

c. Understanding techniques and strategies for dealing with suicidal patients[22]

d. Understanding techniques and strategies for dealing with aggression (patients/families/other) [22]

e. Family dynamics (e.g. mediating, dealing with conflict[10]

11. RELIGION AND SPIRITUALITY

a. Understanding the difference between religious and spiritual needs[6, 39]

b. Understanding and acceptance of, and respect for, the spiritual needs of persons at the end of life[6, 39]

c. Understanding spiritual diversity[10, 19, 39]

d. Providing religious/spiritual support to patients and their families[6, 10, 24, 39]

12. VOLUNTEER AS PATIENT/FAMILY ADVOCATE

In some instances, volunteers can occupy a 'middle ground' between paid health-care professionals (eg, doctors, nurses) and the patient's family and friends. As such, volunteers occupy a space outside both professional and family roles. Volunteers may become aware of patient/family needs that are not being met, providing opportunity to advocate for those patients, or support families to advocate for themselves.

a. How to provide advocacy support for patients and their families[7, 11, 23, 36, 40, 41]

b. Understanding patient rights[23]

c. Being a source of informational support to patients and their families[6]

TRAINING PROGRAMME INFRASTRUCTURE: ISSUES OF RESPONSIBILITY TO THE VOLUNTEER AND THE CARE PROVIDING ORGANISATION

The following categories highlight organisational issues related to setting up a volunteer service and embedding it into the organisational structure.

13. VOLUNTEER RECRUITMENT/RETENTION

a. Use of 'motivation' (to be a volunteer) assessment tool as part of the volunteer selection process[30, 42, 43]

b. Use of a ‘personality’ assessment tool as part of the selection process[43-45]

14. VOLUNTEER SUPPORT

a. Self-care information and strategies and personal resilience[11, 19, 30, 32, 37, 41, 46-48]

b. Regular ongoing mentoring[3, 8, 10, 16, 17, 21, 29, 32, 33, 36, 41, 49]

c. Personal Death Awareness[22, 50]

d. Rituals in dying: practicing 'rituals' and other ways to honour the lives of patients[10, 49, 51]

e. Establish an environment for informal supervision/formal structured supervision with feedback[21]

f. Coping Strategies for dealing with suffering and death[4, 24, 30, 47, 48]

g. Access to wider support services and Psychological support[47, 48]

h. Training updates and other ongoing educational opportunities[10, 36, 49]

15. COMMUNITY ENGAGEMENT AND ADVOCACY FOR THE VOLUNTEER PROGRAMME

a. How to engage with community outreach opportunities within the local community to raise awareness of the volunteer programme[40]

b. Engaging with staff and management within the care providing organisation, to promote the work of the volunteer service[29]

16. VOLUNTEER COMPETENCY AND VOLUNTEER ASSESSMENT

a. Development of 'Core Competencies' for volunteers providing support to patients in the last days of life, and their families[23, 28]

b. Development/agreement of 'standard' outcome measures to evaluate benefit of the programme[23, 52]

c. Include 'formative Assessment' of volunteers following training programme

d. Include 'Summative Assessment' of volunteers following training programme[5, 53]

17. ISSUES OF ORGANISATIONAL INFRASTRUCTURE AND IMPLEMENTATION

a. Embed the volunteer service within the organisation, with attention to organisational/regional/national/international Legislation affecting volunteers[7, 9, 12, 20, 38, 49]

b. Establish organisational policy and procedures for role of the volunteer service and volunteer coordinator [3, 7, 9, 12, 20, 34, 49, 54]

REFERENCES

1. Goossensen, A., et al., *Defining volunteering in hospice and palliative care in Europe: an EAPC White Paper.* European Journal of Palliative Care, 2016. **23**(4): p. 184-191.
2. Claxton-Oldfield, S., *Hospice palliative care volunteers: the benefits for patients, family caregivers, and the volunteers.* Palliat Support Care, 2015. **13**(3): p. 809-13.
3. Burbeck, R., et al., *Understanding the role of the volunteer in specialist palliative care: a systematic review and thematic synthesis of qualitative studies.* BMC Palliative Care, 2014. **13**(1): p. 3.
4. Germain, A., et al., *The use of reflective diaries in end of life training programmes: a study exploring the impact of self-reflection on the participants in a volunteer training programme.* BMC Palliative Care, 2016. **15**(1): p. 28.
5. Brock, C.M. and C.M. Herndon, *A Survey of Hospice Volunteer Coordinators: Training Methods and Objectives of Current Hospice Volunteer Training Programs.* Am J Hosp Palliat Care, 2017. **34**(5): p. 412-416.
6. Claxton-Oldfield, S. and N. Gosselin, *How Can I Help You? A Study of the Perceived Importance of Different Kinds of Hospice Palliative Care Volunteer Support.* American Journal of Hospice and Palliative Medicine®, 2011. **28**(4): p. 271-275.
7. Vanderstichelen, S., et al., *Palliative care volunteerism across the healthcare system: A survey study.* Palliat Med, 2018. **32**(7): p. 1233-1245.
8. Burbeck, R., et al., *Volunteers in specialist palliative care: a survey of adult services in the United Kingdom.* Journal of palliative medicine, 2014. **17**(5): p. 568-574.
9. Morris, S., et al., *A narrative literature review of the contribution of volunteers in end-of-life care services.* Palliative Medicine, 2013. **27**(5): p. 428-436.
10. Wittenberg-Lyles, E., G. Schneider, and D.P. Oliver, *Results from the national hospice volunteer training survey.* J Palliat Med, 2010. **13**(3): p. 261-5.
11. Pesut, B., et al., *Promoting volunteer capacity in hospice palliative care: a narrative review.* Am J Hosp Palliat Care, 2014. **31**(1): p. 69-78.
12. Claxton-Oldfield, S., N. Gosselin, and J. Claxton-Oldfield, *Imagine You Are Dying: Would You Be Interested in Having a Hospice Palliative Care Volunteer?* American Journal of Hospice and Palliative Medicine, 2009. **26**(1): p. 47-51.
13. Dodd, S., et al., *'Being with' or 'doing for'? How the role of an end-of-life volunteer befriender can impact patient wellbeing: interviews from a multiple qualitative case study (ELSA).* Support Care Cancer, 2018. **26**(9): p. 3163-3172.
14. McKee, M., et al., *It takes a whole community: the contribution of rural hospice volunteers to whole-person palliative care.* J Palliat Care, 2010. **26**(2): p. 103-11.
15. Claxton-Oldfield, S. and A. Bhatt, *Is There a Place for Humor in Hospice Palliative Care? Volunteers Say "Yes"!* Am J Hosp Palliat Care, 2017. **34**(5): p. 417-422.
16. Planalp, S. and M. Trost, *Motivations of Hospice Volunteers.* American Journal of Hospice and Palliative Medicine®, 2009. **26**(3): p. 188-192.
17. Vanderstichelen, S., et al., *The liminal space palliative care volunteers occupy and their roles within it: a qualitative study.* BMJ Support Palliat Care, 2020. **10**(3): p. e28.
18. Delaloye, S., et al., *Volunteers trained in palliative care at the hospital: an original and dynamic resource.* Palliat Support Care, 2015. **13**(3): p. 601-7.
19. Brighton, L.J., et al., *'End of life could be on any ward really': A qualitative study of hospital volunteers' end-of-life care training needs and learning preferences.* Palliative Medicine, 2017. **31**(9): p. 842-852.
20. Rajashree, K., *Training programs in communication skills for health care professionals and volunteers.* Indian journal of palliative care, 2011. **17**(Suppl): p. S12-S13.
21. Somes, E., et al., *Perceptions of trained laypersons in end-of-life or advance care planning conversations: a qualitative meta-synthesis.* BMC Palliat Care, 2018. **17**(1): p. 98.

22. Deodhar, J.K. and M.A. Muckaden, *Continuing professional development for volunteers working in palliative care in a tertiary care cancer institute in India: a cross-sectional observational study of educational needs.* Indian J Palliat Care, 2015. **21**(2): p. 158-63.
23. Gerber, P.S., *Last watch: developing an inpatient palliative volunteer program for U.s. Veterans in hospice.* Omega (Westport), 2013. **67**(1-2): p. 87-95.
24. Pawłowski, L., et al., *Nationwide survey on volunteers' training in hospice and palliative care in Poland.* BMJ Support Palliat Care, 2019. **9**(3): p. e25.
25. Jovanovic, M., *Cultural Competency and Diversity Among Hospice Palliative Care Volunteers.* American Journal of Hospice and Palliative Medicine®, 2012. **29**(3): p. 165-170.
26. Jovanovic, M., *Improving Cultural Competency Among Hospice and Palliative Care Volunteers:Recommendations for Social Policy.* American Journal of Hospice and Palliative Medicine®, 2012. **29**(4): p. 268-278.
27. Claxton-Oldfield, S., S. Guigne, and J. Claxton-Oldfield, *How to attract more males to community-based hospice palliative care volunteer programs.* Am J Hosp Palliat Care, 2009. **26**(6): p. 439-48.
28. McCallum, M., et al., *Developing a Palliative Care Competency Framework for Health Professionals and Volunteers: The Nova Scotian Experience.* J Palliat Med, 2018. **21**(7): p. 947-955.
29. Söderhamn, U., et al., *To be a trained and supported volunteer in palliative care – a phenomenological study.* BMC Palliative Care, 2017. **16**(1): p. 18.
30. Garbay, M., M.C. Gay, and S. Claxton-Oldfield, *Motivations, Death Anxiety, and Empathy in Hospice Volunteers in France.* Am J Hosp Palliat Care, 2015. **32**(5): p. 521-7.
31. Claxton-Oldfield, S., M. Gallant, and J. Claxton-Oldfield, *The Impact of Unusual End-of-Life Phenomena on Hospice Palliative Care Volunteers and Their Perceived Needs for Training to Respond to Them.* OMEGA - Journal of Death and Dying, 2018. **0**(0): p. 0030222818788238.
32. Phillips, J., L. Andrews, and L. Hickman, *Role Ambiguity, Role Conflict, or Burnout:Are These Areas of Concern for Australian Palliative Care Volunteers? Pilot Study Results.* American Journal of Hospice and Palliative Medicine®, 2014. **31**(7): p. 749-755.
33. Ning, X.H., J. Li, and Y.R. Xiang, *Running a Volunteer Program for Palliative Care in a Chinese Hospital: Our Practice and Experience.* Chin Med Sci J, 2018. **33**(4): p. 216-220.
34. Claxton-Oldfield, S., L. Gibbon, and K. Schmidt-Chamberlain, *When to say "yes" and when to say "no": boundary issues for hospice palliative care volunteers.* Am J Hosp Palliat Care, 2011. **28**(6): p. 429-34.
35. Berry, P. and S. Planalp, *Ethical issues for hospice volunteers.* Am J Hosp Palliat Care, 2008. **25**(6): p. 458-62.
36. Jack, B.A., et al., *'A bridge to the hospice': the impact of a Community Volunteer Programme in Uganda.* Palliat Med, 2011. **25**(7): p. 706-15.
37. Bradas, C., et al., *Implementing the 'No One Dies Alone program': process and lessons learned.* Geriatr Nurs, 2014. **35**(6): p. 471-3.
38. Block, E.M., et al., *Got volunteers? Association of hospice use of volunteers with bereaved family members' overall rating of the quality of end-of-life care.* J Pain Symptom Manage, 2010. **39**(3): p. 502-6.
39. Gratz, M., et al., *Spiritual care in the training of hospice volunteers in Germany.* Palliative and Supportive Care, 2016. **14**(5): p. 532-540.
40. Claxton-Oldfield, S. and K. Blacklock, *Hospice Palliative Care Volunteers as Program and Patient/Family Advocates.* Am J Hosp Palliat Care, 2017. **34**(9): p. 844-848.
41. Brown, M.V., *How they cope: a qualitative study of the coping skills of hospice volunteers.* Am J Hosp Palliat Care, 2011. **28**(6): p. 398-402.
42. Claxton-Oldfield, S., et al., *A Study of the Motivations of British Hospice Volunteers.* American Journal of Hospice and Palliative Medicine®, 2013. **30**(6): p. 579-586.

43. Stelzer, E.-M. and F.R. Lang, *Motivations of German Hospice Volunteers:How Do They Compare to Nonhospice Volunteers and US Hospice Volunteers?* American Journal of Hospice and Palliative Medicine®, 2016. **33**(2): p. 154-163.
44. Claxton-Oldfield, S. and Y. Banzen, *Personality characteristics of hospice palliative care volunteers: the "big five" and empathy.* Am J Hosp Palliat Care, 2010. **27**(6): p. 407-12.
45. Claxton-Oldfield, S., J. Claxton-Oldfield, and S. Paulovic, *Personality traits of British hospice volunteers.* Am J Hosp Palliat Care, 2013. **30**(7): p. 690-5.
46. Brown, M.V., *The stresses of hospice volunteer work.* Am J Hosp Palliat Care, 2011. **28**(3): p. 188-92.
47. Pöyhiä, R., et al., *Palliative Care Volunteers Have High Workload but No Burnout: A Questionnaire Survey from Tanzania.* Journal of Palliative Medicine. **0**(0): p. null.
48. Claxton-Oldfield, S., *Hospice Palliative Care Volunteers:A Review of Commonly Encountered Stressors, How They Cope With them, and Implications for Volunteer Training/Management.* American Journal of Hospice and Palliative Medicine®, 2016. **33**(2): p. 201-204.
49. Apenteng, B.A., et al., *Trends in the Use of Volunteers in US Hospices: 2000 to 2010.* American Journal of Hospice and Palliative Medicine, 2016. **33**(3): p. 256-263.
50. Nissim, R., et al., *Motivations, Satisfaction, and Fears of Death and Dying in Residential Hospice Volunteers:A Prospective Longitudinal Study.* American Journal of Hospice and Palliative Medicine®, 2016. **33**(4): p. 335-339.
51. Montross-Thomas, L.P., et al., *Personally Meaningful Rituals: A Way to Increase Compassion and Decrease Burnout among Hospice Staff and Volunteers.* J Palliat Med, 2016. **19**(10): p. 1043-1050.
52. Horey, D., et al., *Training and supportive programs for palliative care volunteers in community settings.* Cochrane Database of Systematic Reviews, 2015(7).
53. Szeliga, M. and J. Mirecka, *Optimization of Methods Verifying Volunteers' Ability to Provide Hospice Care.* J Cancer Educ, 2018. **33**(2): p. 470-476.
54. Tao, X. and X.H. Ning, *[Survey on the Accompanying Experience of Palliative Hospice Volunteers in Peking Union Medical College Hospital].* Zhongguo Yi Xue Ke Xue Yuan Xue Bao, 2018. **40**(3): p. 395-400.
