# Supplementary material for: ILIVE Project Volunteer study. Developing international consensus for a European Core Curriculum for hospital end-of-life-care volunteer services, to train volunteers to support patients in the last weeks of life: A Delphi study
Source: Palliat Med. 2021 Oct 20;36(4):652–70. doi: 10.1177/02692163211045305 (PMC9006393; doi:10.1177/02692163211045305)
Supplement: sj-pdf-3-pmj-10.1177_02692163211045305 – Supplemental material for ILIVE Project Volunteer study. Developing international consensus for a European Core Curriculum for hospital end-of-life-care volunteer services, to train volunteers to support patients in the last weeks of life: A Delphi study [file sj-pdf-3-pmj-10.1177_02692163211045305.pdf]

Scoping Review Protocol

REVIEW QUESTION

What are the key concepts/themes related to voluntary palliative care provision, roles, education, training, organisational issues, supervision and support?

REVIEW OBJECTIVE

Identified key concepts/themes will be used to develop items for inclusion on a Delphi questionnaire.

REVIEW PROTOCOL

	Parameters	Inclusion Criteria	Exclusion Criteria
INCLUSION / EXCLUSION CRITERIA	**Location**	*Anywhere*	*None*
	**Language** (STEP 1)	*Articles written in English*	*Articles **not** written in English*
	**Time Frame** (STEP 1)	*Articles published Between January 2009 and February 2019*	*Articles published before January 2009*
	**Population** (STEP 1, 2 3,4)	*Articles concerning volunteers (according to OPCARE9 definitions)*<br><br>*Articles that are concerned with palliative care, care at the end of life, or care in the last days of life.*<br><br>*Articles concerning palliative care issues that remain unchanged into last days of life.*	*Articles concerning family carers*<br><br>*Articles that are **not** concerned with palliative care, care at the end of life, or care in the last days of life.*<br><br>*Articles relating to children.*<br><br>*Articles relating to voluntary work by health care professionals*

	**Study Type**	*All Types: Primary Research, Book reviews, opinion pieces, literature reviews, policy documents, Educational Curricula, Editorials, Letters etc*	

INCLUSION / EXCLUSION METHODOLOGY

STEP 1: Search String

SEARCH TERMS

hospice OR "end of life" OR "terminally ill" OR "terminal illness" OR "terminal care" OR palliat* OR "dying patient" OR "approaching death" OR "advanced cancer" OR "terminal cancer"	**[ANYWHERE/ALL TEXT]**
AND (volunt* OR "informal care*")	**[ANYWHERE/ALL TEXT]**
NOT cell	**[Title/Abstract] or Keywords**
NOT euthanasia	**[Title/Abstract] or Keywords**
NOT dementia	**[Title/Abstract] or Keywords**
NOT apoptosis	**[ANYWHERE/ALL TEXT]**

LIMITS

ENGLISH	**LANGUAGE**
Jan 2009 – Feb 2019	**PUBLICATION DATE**
When possible:	**LIMIT TO HUMANS ONLY**<br>**LIMIT TO ADULTS ONLY**

STEP 2: Remove articles by title based on relevance to volunteer issues in palliative/EOL care

Is the article title concerned with palliative care, care at the end of life, or care in the last days of life?

YES / UNKNOWN ↓ *NO* → ***EXCLUDED***

STEP 3: Remove articles by abstract based on relevance to volunteer issues in palliative/EOL care

YES / UNKNOWN ↓ *NO* → ***EXCLUDED***

		**Inclusion Criteria**	**Exclusion Criteria**
	**Article Outcome / Content (STEP 4)**	*Articles that are related to voluntary palliative care provision, roles, education, training, organisational issues and supervision, support.*	*Articles that ARE NOT related to voluntary palliative care provision, roles or education, training, organisational issues and supervision, support.*<br><br>Supplementary criteria:<br><br>*Articles that describe 'bereaved carers'*<br><br>*Articles relating to 'teen volunteers'*

STEP 4: Remove articles by full text based on relevance to volunteer care provision, roles, education, training, organisational issues and supervision, support. in palliative/EOL care

YES ↓ *NO* → ***EXCLUDED***

		Inclusion Criteria	Exclusion Criteria
	**Article Outcome / Content (STEP 4)**	*Articles that are related to voluntary palliative care provision, roles, education, training, organisational issues and supervision, support.*	*Articles that ARE NOT related to voluntary palliative care provision, roles or education, training, organizational issues and supervision, support.*<br><br>Supplementary criteria:<br><br>*Articles that describe 'bereaved carers' i.e. family or close friends*<br><br>*Articles relating to 'teen volunteers'*<br><br>*If reference is a conference poster or abstract with no accompanying full text*

STEP 5: Review content of articles.

Articles will be coded to develop a thematic framework, based on the main outcomes/discussion reported.

Sections of article text that are related to the review question will be coded. The purpose of the codes will be to label the text so that salient information related to the review question can be identified. Codes will aim to identify key characteristics or factors related to service implementation, education and training of volunteers. Codes will then be reviewed, and the coded text will be categorised to create themes.
