# Supplementary material for: ILIVE Project Volunteer study. Developing international consensus for a European Core Curriculum for hospital end-of-life-care volunteer services, to train volunteers to support patients in the last weeks of life: A Delphi study
Source: Palliat Med. 2021 Oct 20;36(4):652–70. doi: 10.1177/02692163211045305 (PMC9006393; doi:10.1177/02692163211045305)
Supplement: sj-pdf-4-pmj-10.1177_02692163211045305 – Supplemental material for ILIVE Project Volunteer study. Developing international consensus for a European Core Curriculum for hospital end-of-life-care volunteer services, to train volunteers to support patients in the last weeks of life: A Delphi study [file sj-pdf-4-pmj-10.1177_02692163211045305.pdf]

TITLE PAGE

Title: iLVE Project Volunteer Study. Developing international consensus for a European Core Curriculum for hospital end-of-life-care volunteer services, to train volunteers to support patients in the last weeks of life: a Delphi study.

Authors:

Tamsin McGlinchey[1], Stephen R Mason[1], Ruthmarijke Smeding[1], Anne Goosensen[2], Inmaculada Ruiz-Torreras[3], Dagny Faksvåg Haugen[4,5], Miša Bakan[6] and John E Ellershaw[1], *on behalf of the iLIVE Consortium*

1. Palliative Care Unit, University of Liverpool, UK
2. University of Humanistic Studies, Netherlands
3. Cudeca Hospice Foundation, Spain
4. Department of Clinical Medicine K1, University of Bergen, Norway
5. Regional Centre of Excellence for Palliative Care, Haukeland University Hospital, Norway
6. University Clinic of Pulmonary and Allergic Diseases Golnik, Slovenia

Corresponding Author:

Tamsin McGlinchey, Palliative Care Unit, University of Liverpool, 200 London Road, Liverpool L3 9TA, UK
Email: Tamsin.mcglinchey@liverpool.ac.uk
Telephone: +44 (0)151 794 8840
